# Respiratory Virus Infections in Hematopoietic Cell Transplant Recipients

**DOI:** 10.3389/fmicb.2018.03294

**Published:** 2019-01-09

**Authors:** Cécile Pochon, Sebastian Voigt

**Affiliations:** ^1^Allogeneic Hematopoietic Stem Cell Transplantation Unit, Department of Pediatric Oncohematology, Nancy University Hospital, Vandœuvre-lès-Nancy, France; ^2^Department of Pediatric Oncology/Hematology/Stem Cell Transplantation, Charité – Universitätsmedizin Berlin, Berlin, Germany; ^3^Department of Infectious Diseases, Robert Koch Institute, Berlin, Germany

**Keywords:** hematopoietic cell transplantation, respiratory virus infection, co-infection, immunosuppression, antiviral therapy, investigational drugs, infection outcome

## Abstract

Highly immunocompromised pediatric and adult hematopoietic cell transplant (HCT) recipients frequently experience respiratory infections caused by viruses that are less virulent in immunocompetent individuals. Most of these infections, with the exception of rhinovirus as well as adenovirus and parainfluenza virus in tropical areas, are seasonal variable and occur before and after HCT. Infectious disease management includes sampling of respiratory specimens from nasopharyngeal washes or swabs as well as sputum and tracheal or tracheobronchial lavages. These are subjected to improved diagnostic tools including multiplex PCR assays that are routinely used allowing for expedient detection of all respiratory viruses. Disease progression along with high mortality is frequently associated with respiratory syncytial virus, parainfluenza virus, influenza virus, and metapneumovirus infections. In this review, we discuss clinical findings and the appropriate use of diagnostic measures. Additionally, we also discuss treatment options and suggest new drug formulations that might prove useful in treating respiratory viral infections. Finally, we shed light on the role of the state of immune reconstitution and on the use of immunosuppressive drugs on the outcome of infection.

## Respiratory Virus Infections in Pediatric and Adult Allogeneic Hematopoietic Cell Transplant Recipients

In early prospective studies evaluating the incidence of respiratory viral infections in allogeneic HCT recipients conducted by two members of the European Group for Blood and Marrow Transplantation (EBMT), frequencies of these infections were determined at 4 and 7.1%, respectively. Furthermore, the highest mortality rates from fatal pneumonia were found with RSV of 60% and AdV of 75% (Ljungman, 1997). Today, 20 years on, these continue to be frequent infections and still contribute to significant mortality with rates ranging between 10 and 50% if infection progresses to the lower respiratory tract (Boeckh, 2008; Englund et al., 2011; Renaud and Campbell, 2011; Protheroe et al., 2012; Chemaly et al., 2014; Spahr et al., 2018).

Human pathogenic viruses frequently causing respiratory infections in the allogeneic HCT setting include RSV, AdV, IFV, PIV, HMPV, RhV, HCoV, and HBoV (Renaud and Englund, 2012; Abbas et al., 2017). Such respiratory virus infections contribute to morbidity and mortality for a number of reasons. Firstly, HCT recipients suffer from prolonged immunosuppression and a lack of both humoral and T cell-mediated immunity impairs viral clearance (Kohlmeier and Woodland, 2009; Schmidt and Varga, 2018). In addition, these patients are usually antibody-depleted and the strength of the conditioning regimen and the ongoing immunosuppressive therapy diminishes the antiviral immune response further. Moreover, the type of graft may have an impact on the immune response and pediatric HCT recipients may also be immunologically naïve because of their age. Secondly, viral factors shape the outcome. Respiratory viruses vary in their pathogenicity, i.e., they cause more severe disease compared with other viruses. Also, there is the capacity for certain strains of a given respiratory virus to display increased virulence as is the case with IFV and it is therefore important to identify viral and host genes that determine virulence. Thirdly, a limited number of effective antivirals or the emergence of drug resistance also hamper successful treatment. Finally, factors like persisting infections, co-infections and co-morbidities will also influence the outcome.

In contrast to immunocompetent individuals, HCT recipients present with prolonged viral shedding and have a higher rate of progression from URI to LRI (Chemaly et al., 2014; de Lima et al., 2014; Kim et al., 2014). Increased viral loads and protracted infections raise the likelihood of progression that differs between viruses. URI has been commonly defined as laboratory-confirmed viral nasal wash or swab with pharyngitis, cough, otitis, nasal discharge and/or congestion in the absence of infiltrates on chest x-ray or computed tomography (CT) scan and hypoxemia, and LRI has been defined as laboratory-confirmed viral nasal wash or swab in the presence of infiltrates on chest x-ray or CT scan suggestive of a viral respiratory infection but not necessarily detection of viral nucleic acid in samples obtained from BAL (Shah et al., 2014). In HCT recipients, RSV is most frequently detected, followed by HMPV and PIV. In 5–50% of cases, these viruses progress to LRI, often complicate the transplant course by causing airflow obstruction or bronchiolitis obliterans and also increase the rate of mortality up to 50% (Chemaly et al., 2014). Several respiratory viruses share a number of risk factors associated with LRI occurrence. It is worth noting that CMV also represents an opportunistic pathogen that is causally related to pneumonia and LRI. However, CMV will not be discussed in this review since it is not primarily considered a respiratory virus. That said, CMV is a pathogen that frequently occurs as part of a simultaneous infection – indeed a respiratory viral infection can often precede bacterial and fungal infections – thus further complications associated with respiratory virus infections need to be acknowledged.

In this review, we will focus on respiratory virus infections and, particularly, those that are commonly detected after HCT. We highlight risk factors that facilitate progression from URI to LRI and discuss diagnostic approaches. Current treatment strategies along with new treatment options will be discussed.

### Adenovirus

Adenovirus is a member of the *adenoviridae* that circulates throughout the year. Currently, 90 human types are known which are further divided into seven species A–G^1^ (accessed on 20 August 2018). Alongside conjunctivitis and diarrhea, AdV can cause pharyngitis, bronchitis and pneumonia but also lethal hepatitis or severe bloody colitis. Pertinent to this review, AdV is a pathogen associated with severe complications in immunosuppressed pediatric HCT recipients including increased mortality (Leen et al., 2006; Feuchtinger et al., 2007; Lion, 2014; Feucht et al., 2015; Hiwarkar et al., 2018). In adult patients, AdV infections are less commonly reported. However, it is possible this perception might be biased by reduced frequency of screening in adults. A study in adult allogeneic HCT recipients reported an infection rate of 2.5%. Pneumonia occurred in 24% of cases and was the most common cause of death associated with AdV (Yilmaz et al., 2013). An important consideration is that AdV infections infrequently present with respiratory symptoms at the onset of infection; instead they are commonly detected by monitoring stool (Lo et al., 2013; Lion, 2014). Indeed, gastrointestinal shedding pre-transplant has been demonstrated to reflect increased risk of viremia after HCT (Kosulin et al., 2018a).

### Human Bocavirus

Human bocavirus was identified in 2005 as a human pathogen that causes respiratory tract infections in infants. It has been assigned to the *parvoviridae* and received its name because of sequence homology to two other members in the genus *Bocaparvovirus*, Bovine parvovirus infecting cattle and Canine minute virus infecting dogs (Allander et al., 2005). Currently, four HBoV variants have been described. Like HBoV3, HBoV1 can enter an episomal state, allowing the establishment of a persistent infection (Kapoor et al., 2011). Whereas HBoV2, HBoV3, and HBoV4 cause gastroenteritis and are genetically diverse, HBoV1 exhibits a stronger association with LRI than enteritis, often in conjunction with other respiratory pathogens, and has limited genetic diversity although reinfections with different variants occur (Arden et al., 2006; Ditt et al., 2008; Arthur et al., 2009; Kapoor et al., 2010; Wang et al., 2010; Martin et al., 2015). Symptoms following infection include cough, dyspnea, pharyngitis, bronchitis, pneumonia, and diarrhea. Several reports have suggested that HBoV1 can cause severe infections on its own and is not a mere bystander virus (Schildgen et al., 2008; Ursic et al., 2011; Edner et al., 2012). Likewise, HBoV can cause life-threatening infections and prolonged shedding in immunocompromised patients, and for immunocompetent children a median shedding time of 50 days has been reported (Kupfer et al., 2006; Allander, 2008; Koskenvuo et al., 2008; de Vries et al., 2009; Martin et al., 2015). This long shedding period might lead to more severe disease in immunocompromised children (Schenk et al., 2007). Importantly, HBoV does not exhibit seasonality and thus remains a threat throughout the year.

### Human Coronavirus

Human coronavirus belongs to the *coronaviridae* that are endemic in humans. Annually HCoV are responsible for 15–30% of URI with pharyngitis and rhinitis in immunocompetent hosts. Historically, two common HCoV were known: HCoV-229E and HCoV-OC43. However, the emergence of Severe Acute Respiratory Syndrome-Coronavirus (SARS-CoV) along with two further HCoV (HCoV-HKU1 and HCoV-NL63) has expanded the family (van der Hoek et al., 2004; Woo et al., 2005). In contrast to the low incidence of bronchitis or pneumonia in healthy children, severe clinical features have been described in immunocompromised patients. Both the presence of a respiratory co-pathogen (RSV) and host factors like young age < 5 years and an immunocompromised status were reported to contribute to LRI. However, it should be noted that only 10 children with HCT were included (Ogimi et al., 2017b, 2018a). In separate studies, HCoV have been associated with increased mortality and prolonged shedding in the HCT setting (Milano et al., 2010; Renaud and Campbell, 2011). Risk factors for prolonged shedding (at least 21 days) in the upper respiratory tract were determined in a cohort of 44 patients and included high viral load, myeloablative conditioning, and prior high-dose steroid use (Ogimi et al., 2017a). Of 44 patients, 31 samples were analyzed shown to contain evidence of HCoV-OC43 (35%), HCoV-NL63 (32%), HCoV-HKU1 (19%), and HCoV-229E (13%) infection. Analysis for duration of shedding showed that none of the strains appeared to cause longer shedding compared with others. In addition, genomic approaches investigated whether viral genome evolution could identify genetic changes associated with prolonged shedding. Identification of such changes could aid the development of new antiviral agents. Single nucleotide polymorphisms could not be identified until day thirty after the onset of viral shedding. This finding might not be surprising given the protracted evolution rate of HCoV. However, overt viral genome changes might occur at a later time point, and changes in genome composition might result in a modification of the treatment strategy (Ogimi et al., 2017a). In another study, HCoV were examined in BAL (Ogimi et al., 2017b). The median time to HCoV LRI occurrence was 302 days after HCT. Among 23 BAL samples analyzed, 48% were HCoV-OC43, HCoV-NL63 was detected in 22%, HCoV-229E in 17% and HCoV-HKU1 in 13%. Although somewhat limited because of sample size, these data show a similar frequency and order of detected viral strains compared to those detected from nasal samples with the exception of HCoV-HKU1 which was predominant in nasal samples (Ogimi et al., 2017a). The detection of RSVs did not change clinical outcome.

### Influenza Virus

Influenza virus, an *orthomyxoviridae* member, exhibits high genomic variability due to an absence of proofreading activity in its RNA – a process that contributes to antigenic drift. As some IFV strains are more virulent than others, the strain of IFV impacts on severity, depending on circulating strains, and this can be impacted upon by the composition of the vaccine and also the uptake of vaccine in the population. Individuals with a compromised immune system such as the elderly and HCT recipients are at increased risk of complications and death, and outbreaks in HCT units have been reported (Lalayanni et al., 2010; Suyani et al., 2011). In HCT recipients, fever maybe absent in about 19% of cases as might classical symptoms such as sore throat, nasal congestion and discharge, cough, chills and myalgia (Ljungman et al., 2011). As with other respiratory viruses, IFV shedding in HCT patients has been reported to be prolonged with a median of 12 days; lymphopenia correlated with the duration of shedding, and steroid use > 1 mg/kg increased IFV secretion (Khanna et al., 2009; Boudreault et al., 2011; Engelhard et al., 2013). Infants may present with sepsis-like symptoms and pneumonia. Complications in adults include pneumonia, myocarditis, encephalitis and Guillain-Barré syndrome. As with PIV infections (see below), bacterial and fungal co-infections with IFV are known (Nichols et al., 2004b; Engelhard et al., 2013). Disease progression to LRI takes place in up to 35% and mortality ranges between 15 and 28% in patients diagnosed with pneumonia. Therefore, early treatment within 2 days of presenting with symptoms has been favored (Whimbey et al., 1994; Nichols et al., 2004b; Chemaly et al., 2006; Kmeid et al., 2016). While risk factors have been validated for RSV and PIV, equivalent quality indicators are limited for IFV. Potential risk factors that might result in LRI are lymphopenia and neutropenia as well as increased age (>65 years) while steroid use is unclear (Ljungman et al., 2001; Chemaly et al., 2006; Choi et al., 2012). IFV genome detection in blood was associated with hypoxemia, respiratory failure, and overall mortality (Choi et al., 2012). Kmeid et al. (2016) applied an ISI that had been developed for RSV to identify patients with IFV infection who might run at risk to develop progression to LRI (Shah et al., 2014). According to variables such as age, neutrophil and lymphocyte counts, conditioning regimen, steroid use and time from transplantation, HCT recipients were grouped into low, moderate, and high risk immunodeficiency categories. A high risk score was applied if the score was between 7 and 12 and was likely to include ANC < 500/mm^3^ (score 3) and/or ALC < 200/mm^3^ (3), age ≥ 40 years (2), myeloablative regimen (1), GvHD (1), steroid use within the last 30 days (1) and recent (within the last 30 days) or pre-engraftment allogeneic HCT (1). Patients with a high risk score had a significantly higher probability of developing LRI compared to the low risk group (Kmeid et al., 2016).

### Human Metapneumovirus

Human metapneumovirus, formerly a member of the *paramyxoviridae* now belonging to the *pneumoviridae*, was identified in 2001 as an agent capable of causing respiratory disease in children (van den Hoogen et al., 2001). In immunocompetent individuals, HMPV causes symptomatic URI and LRI predominantly in children or adults above 65 years of age (Boivin et al., 2002). No specific symptoms that distinguish HMPV from other viral respiratory infections exist. It has been reported that disease is more severe in case of a RSV co-infection but these data are considered controversial. In immunocompetent children, one study revealed that HMPV with RSV co-infection increased bronchiolitis (Semple et al., 2005), however, this could not be substantiated in other studies that investigated single infections and co-infections for HMPV and RSV (Moe et al., 2017; Yan et al., 2017). For both HMPV and RSV, A and B types occur that can be distinguished by different primer sets in PCR (Renaud et al., 2013). Immunocompromised children are at higher risk of developing LRI with higher risk of requiring intensive care treatment with increased mortality (Chu et al., 2014). In a study of 21 HCT recipients, HPMV infection was documented in the majority of patients but did not cause any symptoms or disease (Debiaggi et al., 2006). However, in a study that included 251 episodes of URI and LRI, 16 of the 22 episodes in which HPMV was detected occurred in HCT recipients, resulting in an infection rate of approximately 6% of all HCT patients (Williams et al., 2005). Another study revealed HPMV in BAL samples from 5 of 163 patients who became symptomatic within a month after engraftment, and fatality reached up to 80% if BAL was positive for HMPV (Englund et al., 2006). Progression to LRI has been reported to take place between 21 and 40% (Renaud and Campbell, 2011). For HMPV, risk factors for mortality include length between HCT and infection; neutropenia, lymphopenia, low monocyte count at diagnosis, and a steroid dose before diagnosis of ≥1 mg/kg (Seo et al., 2016). In the latter study, the presence of co-pathogens contributed to higher risk. Glucocorticoid application and lymphopenia have been identified as risk factors that contribute to progression from URI to LRI (Seo et al., 2016). In a systematic review, HMPV progressed to LRI in 34% of cases and the mortality rate increased from 6 to 27% when LRI was present but no HMPV-associated risk factors for increased mortality could be identified (Shah et al., 2016b).

### Parainfluenza Virus

Parainfluenza virus is a member of the *paramyxoviridae* that circulates throughout the year. PIV causes LRI in approximately 20% of healthy children (Bicer et al., 2013) and is also responsible for life-threatening LRI in HCT recipients (Srinivasan et al., 2011; Shah et al., 2016a). Four PIV serotypes are known. PIV serotype 3 (PIV-3) is the most frequent occurring serotype and has been responsible for infections after HCT (Cortez et al., 2001; Nichols et al., 2004a; Maziarz et al., 2010; Hodson et al., 2011). In one study comprising 3577 patients with PIV infection, 6.4% had an infection with PIV-3, and 24% of these patients developed pneumonia (Nichols et al., 2001a). Another study reported PIV infections in up to 18% of cases during the first 3 months post HCT (Peck et al., 2007). In 20–40% of patients who experience URI, infection progresses to LRI with a median time of 78 days. Virus-associated mortality is around 10% after PIV infection but increases to 27% if infection progresses to LRI (Nichols et al., 2001a; Srinivasan et al., 2011; Chemaly et al., 2012; Ustun et al., 2012; Seo et al., 2014a; Shah et al., 2016a). PIV risk factors for progression to LRI are lymphopenia, steroid use and co-infections with other respiratory agents, however, myeloablation and infections in the early post-transplant period were variables with unclear association (Seo et al., 2014b; Shah et al., 2016a). Pulmonary co-pathogens, e.g., *Aspergillus fumigatus*, are often present in patients with pneumonia and highly contribute to mortality (Nichols et al., 2001a; Ustun et al., 2012).

### Respiratory Syncytial Virus

Respiratory syncytial virus, now also belonging to the *pneumoviridae*, is a frequent pathogen in infants and children that causes URI with rhinitis and laryngitis but also bronchitis and pneumonia if progression to LRI occurs. In HCT recipients, RSV infections occur up to 30% (Martino et al., 2005; Khanna et al., 2008; Avetisyan et al., 2009; Shah and Chemaly, 2011; Waghmare et al., 2013). In approximately half of the HCT recipients with URI caused by RSV, infection will progress and result in pneumonia that is associated with average mortality rates around 30% without effective treatment (Nichols et al., 2001b; Boeckh et al., 2007; Khanna et al., 2008; Shah and Chemaly, 2011; Seo et al., 2013; Waghmare et al., 2013). Risk factors for progression to LRI include advanced age, lymphopenia, myeloablative regimen, steroid use, GvHD and pre-engraftment infection and occur at a mean of 38% (Nichols et al., 2001b; Martino et al., 2005; Khanna et al., 2008; Shah and Chemaly, 2011; Hirsch et al., 2013). Further, smoking history, conditioning with high-dose total body irradiation with 1200–1575 cGy and specifically an absolute lymphocyte count ≤ 100/mm^3^ at URI onset were significantly associated with progression to LRI. In contrast, lung function, steroid use, lymphocyte engraftment dynamics, RSV subtypes and subtype-specific neutralizing antibody levels were not associated with progression, revealing transplant-related rather than viral factors which might put a focus on the characterization of RSV-specific T cells (Kim et al., 2014). In addition, absolute lymphocyte counts of > 1000/mm^3^ at URI onset were protective and no advance to LRI was observed. Another study that examined risk factors for RSV RNA detection in serum or plasma identified mechanical ventilation, neutropenia, monocytopenia as well as thrombocytopenia as associated factors, however, lymphopenia and steroid use did not increase the risk of RSV RNA plasma detection (Waghmare et al., 2013). For that study, a median time of 52 days to LRI onset was described.

As described for IFV, an ISI had been originally proposed to stratify RSV-infected patients into groups based on their risk of progression from URI to LRI and to evaluate RSV-related mortality (Shah et al., 2014). This stratification should help to identify patients who might profit from antiviral therapy such as aerosolized ribavirin. This index is based on age, absolute neutrophil and lymphocyte counts, acute or chronic GvHD, myeloablative conditioning, corticosteroid use and time of infection. However, validation of this index needs to be performed in multicenter studies.

### Rhinovirus

Rhinovirus, a member of the *picornaviridae*, comprises more than 100 serotypes and are divided into the three species A, B and C. There is no seasonal preference for RhV, however, more infections might occur during spring and autumn. RhV are the most frequently detected community-associated respiratory viruses that cause cough, a runny nose but also bronchitis or pneumonia in HCT recipients (Ison et al., 2003; Milano et al., 2010). Symptomatic RhV infections occur in 10–30% of HCT recipients and initial high viral loads are associated with prolonged shedding (Parody et al., 2007; Milano et al., 2010; Ogimi et al., 2018b). A recent study showed that median shedding time among patients with RhV infection was 9.5 days, and 18/38 (47%) of HCT recipients had prolonged RhV shedding (at least 21 days; range, 2–89 days) that was similar for all three species but the majority of patients (69%) shed RhV species A. Risk factors for prolonged RhV shedding pointed to a high initial viral load as defined by a Ct value below the median (30.3) (Ogimi et al., 2018b). Infection control measures should consider these long infection periods. Progression to LRI is increasingly being detected and fatal outcomes due to RhV infections have been described (Gutman et al., 2007; Jacobs et al., 2013; Ogimi et al., 2018b).

## From Colonization to Symptomatic Infection After HCT

Patients who reveal respiratory tract infection symptoms before HCT should be tested for respiratory pathogens by multiplex PCR. Campbell et al. analyzed data from 458 adult and pediatric patients and identified 25% of patients to be positive for at least one respiratory virus pre-transplant. Symptomatic patients had lower survival 100 days after HCT compared with patients with negative test results and increased overall mortality. Since this risk was increased even if only RhV was detected, the authors proposed to consider deferral of HCT in symptomatic patients with any respiratory virus positive, not just only in case of RSV, IFV, PIV, or HMPV detection. Asymptomatic patients with a virus detected did not exhibit higher mortality and HCT might proceed as planned; however, it is unclear if these patients actually shed replicating virus or if only viral nucleic acid is being detected. There was no higher incidence of bronchoscopy in the symptomatic versus the asymptomatic group. Any decision to delay transplant has to consider HCT factors such as underlying disease, the conditioning regimen associated with it and donor logistics (Campbell et al., 2015). In a follow-up study that focused on pediatric HCT recipients, Kim et al. (2017) detected respiratory viruses pre-transplant in 81 of 218 patients. RhV were detected in 24% of cases, followed by a group of viruses consisting of AdV, RSV, HMPV PFV, and PIV (11%). Pre-transplant detection was associated with increased hospitalization during the first 100 days, and HCoV was only detected pre-transplant in 2% of cases.

Since the conditioning regimen might have an impact on the incidence of respiratory virus infections pre-transplant, patients with myeloablative and non-myeloablative conditioning were compared. The incidences for respiratory virus infections were similar among both groups. However, in contrast to patients receiving non-myeloablative conditioning, LRI are significantly increased during the first 100 days post HCT when myeloablative regimen was given (Schiffer et al., 2009).

Immediately after HCT in the neutropenic phase, patients frequently develop febrile episodes and are especially prone to bacterial infections. Therefore, they are often treated with antibiotics to protect them from life-threatening bacterial infections. This treatment alters microbiota composition and has an impact on immune responses (Ichinohe et al., 2011; Abt et al., 2012; Dyer et al., 2016). An immunodulatory compound associated with microbiota is butyrate, a short chain fatty acid that is important in maintaining gut microbiome equilibrium as well as in controlling immune responses in organs such as the lung (Donohoe et al., 2011; Chang et al., 2014). In a recent study, Haak et al. (2018) investigated the influence of butyrate on microbiota composition and its influence on respiratory viral infections and the associated risk to develop LRI in HCT recipients. Patients who received antibiotic treatment resulting in altered microbiota colonization and diminished butyrate-producing bacteria had a higher risk of developing LRI indicating that the presence of butyrate-producing bacteria appears to be protective (Haak et al., 2018). A recent analysis from the Seattle group has shown that antibiotic exposure before the onset of viral respiratory infection increases the risk of progression from URI to LRI in case of HMPV, PIV and RSV infection (Ogimi et al., 2018a). However, a certain class of antibiotics that poses a particular risk could not be identified.

Another recent study described significant lower overall survival for patients with respiratory virus infection accompanied by bacterial co-infection that contributed to increased mortality (Pinana et al., 2018). Risk factors for developing bacterial co-infection following a respiratory viral infection were steroid use ≥ 1mg/kg/d and CMV DNAemia requiring antiviral therapy. Mortality-associated risk factors such as lymphopenia < 0.5 × 109/ml, CMV DNAemia requiring antiviral therapy at the time of viral LRI diagnosis and oxygen support at the time of BAL were used to establish a risk score in order to stratify patients to help predict mortality, no matter if a co-infection was present or not. This new risk score was compared to other scores including the ISI described above, however, the application of ISI criteria did not prove useful to predict mortality in this cohort. Currently, ISI does not include co-infections as a variable, and future studies are needed to validate and compare different risk scores (Pinana et al., 2018). The profound lymphopenia after HCT has been described as a risk factor in the study above and multiple other studies, and it has recently been suggested that CD8 T cells may protect against a secondary infection (Schmidt and Varga, 2018).

## Diagnostic Workup

The fourth European Conference on Infections in Leukemia (ECIL-4) developed guidelines for diagnosis and treatment on several respiratory viruses (Hirsch et al., 2013). HCT candidates and recipients with URI or LRI should be tested for respiratory viruses to guide infection management and possible deferral of transplantation. Specimens should be taken from the site of infection; for URI, pooled swabs should be analyzed and for LRI, a tracheal aspirate or ideally a BAL should be performed. However, patient circumstances often do not permit invasive procedures. These guidelines favored a first-line screening for IFV A and B, RSV, and PIV, however, with more advanced multiplex PCR assays becoming available, all agents might be examined at once (Waghmare et al., 2016).

Over the past years, more respiratory infections in HCT recipients have been reported due to the development and use of new diagnostic methods. Routine molecular diagnostics of respiratory viruses nowadays includes multiplex PCR approaches that have improved detection of respiratory agents in HCT recipients (Leber et al., 2018; Sam et al., 2018). Multiplex assays covering the relevant respiratory viral agents are preferred over laborious and time-consuming viral culture and direct fluorescence antibody assays because of its sensitivity, specificity and rapid turnaround time. In addition, viral nucleic acid quantification is important because determination of a viral load might indicate prolonged viral shedding in case of RhV (Ogimi et al., 2018b). However, this does not provide information about active replication and viral culture techniques are not well established, e.g., for HCoV.

A problem with virus detection is a lack of standardization among assays since different primers, probes and techniques are used (Huang et al., 2017). Nasal respiratory swabs are routinely performed pre-transplant and should be collected in case of suspected respiratory infection. A large prospective study associated respiratory virus detection before transplantation with prolonged hospitalization and decreased survival at day 100 (Campbell et al., 2015). RhV detection after a routine pre-transplant screening can result in fatal infection, even if the patient is asymptomatic and presents with regular findings on chest CT (Milano et al., 2010; Campbell et al., 2015; Waghmare et al., 2016). If any respiratory virus is identified by multiplex PCR pre-transplant, HCT should be postponed (Bredius et al., 2004; Peck et al., 2004; Hirsch et al., 2013). However, as occurs in leukemia relapse cases, the time window from remission to HCT can be narrow, thus making a decision difficult. Irrespective of URI or LRI, transplant should be delayed if possible, probably even in cases where data are scarce as is the case in HCoV and HBoV infections. While episodes of both URI and LRI have shown a higher mortality risk in symptomatic adults in contrast to asymptomatic patients (Abandeh et al., 2013), transplant delay should be recommended in symptomatic adults. However, in pediatric patients who tend to shed virus more frequently than adults and acquire viruses more frequently, transplant delay should be considered on a case by case basis in asymptomatic patients.

In aiding to determine progression from URI to LRI, radiologic signs are considered an important component although inter-observer variability is high and radiologic signs are sometimes difficult to interpret (Elemraid et al., 2014; Franquet, 2018). It is important to perform useful radiologic diagnostics in symptomatic patients with suspected LRI. Chest x-rays are generally not recommended because lack of sensitivity but CT should be performed to evaluate LRI. In children, chest x-rays do not provide specific information in case of HMPV infection where common findings include hyperinflation, atelectasis and perihilar opacities (Hilmes et al., 2017).

In an attempt to try and provide a standardized framework a scoring tool named the RSI has been established to quantify severity of radiologic findings and correlate them with outcome in PIV LRI. Infiltrate expansion, when longitudinally assessed by RSI, was predictive of mortality in patients with PIV-associated disease and discrepant results were obtained when CT and chest X-ray findings were compared. Early signs suggest that the RSI appears to be a useful tool to predict mortality but needs to be validated in future studies (Sheshadri et al., 2018).

Computed tomography findings associated with LRI include bronchial wall thickening and diffuse or patch-like ground glass opacities as a sign of interstitial infiltrates (Franquet et al., 2006; Herbst et al., 2013). However, radiologic approaches often do not show clear signs of infection in symptomatic patients. Additionally, the occurrence of simultaneous infections means correlating a specific virus with CT findings is difficult. To address this, a retrospective study by Kim et al. (2016) examined CT scans from LRI patients with BAL specimens and single infections of either RSV, IFV or PIV in order to identify virus-characteristic CT lesions. Patch consolidations of at least one centimeter or more than one segmental level were only observed in PIV infections. Ground-glass opacities were identified in all CT scans when IFV was detected and were less frequently seen with PIV (detected in 71% of cases) and RSV (67%). Bronchial wall thickening was prominent with IFV and RSV in about two thirds of CT scans analyzed whereas it was seen in only one third in PIV infections. Anatomically, RSV infection was localized in the upper and middle lobes (44%), PIV preferentially in the lower lobes (69%) and IFV infection resulted in a diffuse pattern (Kim et al., 2016). However, due to the retrospective nature of the study by Kim et al., no distinction between early and late infection stage CT findings could be made which might have revealed different patterns. Other studies similarly identified ground-glass opacities for IFV infections (Franquet et al., 2006; Kanne et al., 2007) and bronchial wall thickening combined with nodules and tree-in-bud in RSV infections (Mayer et al., 2014). Another small study also detected bronchial wall thickening in patients with confirmed RSV infections but also ground-glass opacities, and these findings were equally seen in hMPV infections where lesions appeared to be more asymmetrical (Syha et al., 2012). Although CT findings appear to be non-specific, they might help to differentiate between pathogens causing LRI if a thorough diagnostic workup including multiplex PCR with representative specimens is employed to largely rule out simultaneous infections.

## Treatment

### Infection Control

#### Virus Spread Prophylaxis

Infection control measures are of high importance and should be in place in case of suspected or confirmed respiratory virus infection (Boeckh, 2008). Since these viruses are transmitted by close contact with infected individuals or contaminated material, hand hygiene is of utmost importance since nosocomial infections have occurred that must be avoided (Hoellein et al., 2016). The use of universal surgical mask might also be useful to prevent respiratory viral infections after HCT (Sung et al., 2016). Patients with documented respiratory virus infection should be isolated with restricted contact and strict protection measures should be applied (gloves, gowning, masks, eye protection) to visitors and healthcare workers (Hirsch et al., 2013). Since HCT recipients might shed these viruses for a long time because of their impaired immunity, precautions should be taken if a patient is discharged. For example, a patient should be escorted through the outpatient clinic to avoid direct contact with and spread to other patients, and prolonged isolation might be necessary (Tomblyn et al., 2009; Hirsch et al., 2013). This might be especially important for RhV in case high viral loads are detected since shedding time in these patients exceeds 21 days (Ogimi et al., 2018b). However, PIV-3 and HMPV infections might go unrecognized in immunocompetent persons, thus making it difficult to prevent infection (Nichols et al., 2001b; Debiaggi et al., 2007; Shah et al., 2016b; Birger et al., 2018).

#### Reduction of Immunosuppression

Each time a respiratory tract infection is proven after HCT, a reduction of immunosuppressive treatment should be the response. A steroid dose greater than 1 or 2 mg/kg/day has been proven to be an independent risk factor for overall mortality in LRI with RhV (Seo et al., 2017), RSV (Shah et al., 2016a), HMPV, and PIV (Waghmare et al., 2016). However, no correlation between progression or mortality and steroid dose was found for IFV (Waghmare et al., 2016).

### Specific Drugs

#### Adenovirus

Adenovirus pneumonia is usually observed in AdV disseminated disease. Preemptive treatment is recommended as soon as viremia is higher or equal to 1000 copies/ml coupled with lymphocyte counts below 300/mm^3^ and CD4 T lymphocyte counts below 25/mm^3^ (Hiwarkar et al., 2017). This is particularly important in high risk patients and those who received cord blood graft or are under steroid treatment (Lindemans et al., 2010). High AdV levels in stool have also been associated with gastrointestinal AdV disease (Feghoul et al., 2015) – an observation that underpins a current clinical trial addressing the usefulness of a preemptive treatment based on stool AdV detection^2^. So far, no recommendation has been made for AdV detection in upper airways. Specific treatment of AdV pneumonia should be given as early as possible after diagnosis (Neofytos et al., 2007; Lindemans et al., 2010).

Several studies have reported successful Cidofovir treatment of AdV infections in immunocompromised hosts after HCT, combined with withdrawal of immunosuppression (e.g., at 5 mg/kg once every week for 2 weeks, followed by a maintenance dose of 5 mg/kg once every fortnight, or three times weekly at 1 mg/kg) (Lindemans et al., 2010; Wy Ip and Qasim, 2013). Cidofovir inhibits incorporation of deoxycytidine triphosphate into viral DNA by the viral DNA polymerase, leading to viral DNA chain termination. Probenecid co-prescription is useful to prevent or diminish cidofovir nephrotoxicity due to its accumulation in renal tubules. Cidofovir is the current standard of care treatment for AdV infections and diseases. However, cidofovir does not clear the virus in the absence of T cell immune reconstitution (Hiwarkar et al., 2017).

Another novel compound is CMX001 (hexadecyloxypropyl cidofovir, brincidofovir, Chimerix), an orally bioavailable lipid conjugate of cidofovir with good oral bioavailability which achieves higher intracellular levels of active drug compared with cidofovir. Brincidofovir lacks nephrotoxicity making it an attractive alternative. In phase I and phase II trials as well as in retrospective studies, brincidofovir has been shown to be highly efficacious in controlling and clearing adenoviraemia (Grimley et al., 2017; Hiwarkar et al., 2017; Ramsay et al., 2017; Averbuch et al., 2018). Gastrointestinal toxicity is the major side effect observed and might be associated with epithelial apoptosis and crypt injury, as might be observed with GvHD (Detweiler et al., 2018).

Further, a small compound inhibitor named HBX has been reported to be effective against AdV infections. A cellular component important for AdV replication is ubiquitin-specific protease 7 (USP-7), a deubiquitinating enzyme of the ubiquitin proteasome pathway. USP-7 interacts with E1B-55K, a regulator of AdV replication. Since USP-7 promotes AdV replication, it represents a potential drug target because blocking its activity could aid in the treatment of AdV infections (Ching et al., 2013). HBX and a derivative were generated that were able to block AdV (C5 type) replication. With the exception of AdV types A12 and A31, HBX effectively blocked *in vitro* replication of all AdV tested, making it a promising new antiviral candidate (Kosulin et al., 2018b).

For AdV, there is no proven role for ganciclovir, foscarnet or immunoglobulin therapy in immunocompromised patients. Importantly, donor lymphocyte infusions and more recently specific anti AdV cytototoxic T cells should be considered in case of AdV disease after HCT (see below). There is anecdotal evidence of successful treatment of AdV with ribavirin (Schleuning et al., 2004) but most studies have not been supportive; (Lankester et al., 2004) and conclude that cidofovir combined with immunotherapy is more efficient and should be preferred as the first treatment option. Of note, Ribavirin is active *in vitro* on species C isolates (Morfin et al., 2005).

#### HBoV, HCoV, and RhV

There is currently no recommendation of specific antiviral therapy due to the lack of effective agents against these viruses and the lack of clinical trials (Hirsch et al., 2013). Several agents against RhV have been described in the context of immunosuppression: Oral or intranasal pleconaril, a capsid binder, was effective in 2 randomized trials to mildly reduce the duration and severity of colds in immunocompetent adults but its development was halted after FDA rejection partly due to concerns regarding virus resistance (Senior, 2002). Vapendavir is another capsid binder under development^3^ for the treatment of RhV infections of asthmatic adults (Waghmare et al., 2016).

#### Influenza Virus

To avoid transmission of IFV, vaccination of family members, household contacts and HCT recipients is recommended. However, HCT recipients might not mount an adequate immune response if vaccination takes place during immunosuppression. Available antivirals for IFV infections include the M2 ion channel inhibitors such as amantadine that exclusively act on IFV-A. However, neuraminidase inhibitors (NAI) are preferred for prophylaxis and treatment of IFV infections since resistance to M2 inhibitors is frequent (Englund et al., 1998; Fiore et al., 2011; von Lilienfeld-Toal et al., 2016). Post exposure prophylaxis with oral oseltamivir, 75 mg bid for 10 days for adults and children whose weight is above 40 kg, is the current treatment of choice. Oral oseltamivir, inhaled zanamivir and IV peramivir are NAIs that were shown to be effective in HCT recipients (Vu et al., 2007; Tomblyn et al., 2009; Casper et al., 2010; Choi et al., 2011; Engelhard et al., 2013). Treatment within 48 h after the occurrence of symptoms will result in better outcome (Ljungman et al., 2001; Nichols et al., 2004b; Tomblyn et al., 2009; Choi et al., 2011; Engelhard et al., 2013); Although treatment should be initiated as early as possible, protracted and also prolonged treatment have been shown to have favorable effects. The duration of therapy should extend the treatment period recommended for immunocompetent hosts to circumvent reoccurrence and might last for 10 days in HCT recipients. Also, longer therapy in case of unsuccessful clearance might be necessary (Engelhard et al., 2013).

The most commonly described mutation resulting in oseltamivir and peramivir resistance is conferred by the H275Y mutation in influenza A H1N1 that can be treated with inhaled zanamivir (Memoli et al., 2010; Engelhard et al., 2013). In Europe during the 2007–2008 winter season, high resistance rates up to 68% of IFV-A (H1N1) to oseltamivir have been reported (Meijer et al., 2009). New agents with potential efficacy against oseltamivir-resistant viruses are being tested like inhaled laninamivir which is available in Japan, JNJ-63623872, a non-nucleoside inhibitor of the RNA polymerase protein PB2, Nitazoxanide, an antiparasitic agent with activity against IFV, and antibodies (MEDI8852, VIS410) (Shahani et al., 2017).

#### Parainfluenza Virus

In a retrospective analysis of 200 patients including 120 HCT recipients, one-third of patients with PIV infection had lower respiratory tract disease and independent risk factors of progression were neutropenia, APACHE II score ≥ 15, and respiratory co-infections within a month of PIV infection. In this study, treatment with aerosolized ribavirin and/or IVIG did not prevent progression to pneumonia and did not affect duration of illness or survival (Chemaly et al., 2012). A meta-analysis performed in 2016 reinforced this evidence of inefficiency of ribavirin for PIV: indeed, PI-LRI progression was not significantly different in HCT recipients who were treated with ribavirin at URI stage, and PIV-associated mortality rate was slightly higher in patients treated with ribavirin-based therapy at LRI stage than in those who were not treated (Shah et al., 2016a). A recombinant sialidase fusion protein inhibitor named DAS181 has been used to treat severe PIV infections after HCT (Waghmare et al., 2015; Dhakal et al., 2016; Salvatore et al., 2016). DAS181 cleaves the Neu5Ac α(2,3)-Gal and Neu5Ac α(2,6)-Gal sialic acid linkages on the surface of respiratory cells that are used by IFV and PIV for attachment and entry, thereby inhibiting the latter. The largest cohort included 16 patients after HCT, 14 of who received DAS181 for PIV-associated pneumonia but unfortunately lacked a control group (Salvatore et al., 2016). Thirteen out of 16 patients responded to treatment, and the three patients who did not respond had a viral, bacterial, or fungal co-infection, respectively. The drug was well tolerated. PIV loads were recorded in 7 of 16 patients using nasopharyngeal swabs. A > 1 log decrease in PIV load was seen in 5 of 7 patients accompanied by a partial or complete clinical response. High-dose IVIG had no effect on mortality after PIV LRI in a retrospective analysis of 544 patients with PIV infection after HCT (Seo et al., 2014a). Therefore, no specific treatment for PIV infection is currently strongly recommended. However, current ECIL-4 guidelines suggest treatment with aerosolized ribavirin or off-label use with systemic ribavirin. For infections other than PIV and RSV, ribavirin use is not recommended (Hirsch et al., 2013).

#### Human Metapneumovirus

In a study among 105 patients with URI from the Fred Hutchinson Cancer Center (Seo et al., 2016), the probability of progression to LRI within 40 days was 16%, and approximately 75% of the patients with URI who progressed to LRI did so within 2 weeks after URI. Even if several small reports support the use of ribavirin with or without IVIG for HMPV infection, a larger study showed no protective effect of ribavirin to reduce HMPV progression and mortality (Renaud et al., 2013). To date, antiviral therapy is not recommended to cure or to prevent infection progression even in patients at higher risk of HMPV progression. No licensed therapeutics or vaccines exist (Wen and Williams, 2015). Therefore, treatment for HMPV remains so far non-specific and mainly supportive. MAb 338, a monoclonal antibody against a fusion protein of HMPV, demonstrated interesting results in hamster and mice models, and further investigation in clinical trials is warranted (Ulbrandt et al., 2006; Hamelin et al., 2010).

#### Respiratory Syncytial Virus

In 2013, international guidelines recommended aerosolized or systemic (oral or IV) ribavirin with IVIG in patients with RSV URI undergoing allogeneic HCT, allogeneic HCT recipients with risk factors for progression to LRI, and allogeneic HCT patients with LRI (Hirsch et al., 2013). However, the additional benefit of IVIG remains controversial. In a retrospective study that reviewed RSV URI or LRI infections in 280 HCT recipients, a combined IVIG and ribavirin treatment was beneficial (Shah et al., 2013). Further, given significant exponential cost increases in aerosolized ribavirin between 2013 and 2015 and lack of evidence that aerosolized ribavirin is more efficient, early oral ribavirin treatment is currently more frequently considered (Waghmare et al., 2016) and is well tolerated (e.g., 15 mg/kg/day in three divided doses for 10 days) (Gorcea et al., 2017). A clinical trial comparing RSV treatment with oral *versus* inhaled ribavirin is ongoing^4^. However, in France, aerosolized ribavirin is currently not available.

Lymphopenia is a specific risk factor that has been associated with progression to LRTI in several studies. Other risk factors such as total body irradiation, smoking history, stem cell source other than peripheral blood stem cells and oxygen requirement might be considered (Seo et al., 2013; Waghmare et al., 2013). A lower virulence of RSV-B compared to RSV-A has been reported (Kelly et al., 2016). As stated above, the ISI might be helpful to identify patients who would benefit from antiviral therapy (Shah et al., 2016a).

Data from studies with the RSV-specific monoclonal antibody palivizumab have shown controversial efficacy, apart from the fact that it is very costly in adults. In larger studies of HCT recipients with RSV LRI, adjunctive palivizumab did not lead to outcome improvement (Seo et al., 2013; Waghmare et al., 2013). Therefore, RSV-specific monoclonal antibody is not recommended as a treatment option and might be only discussed for very young (age < 2 years) allogeneic HCT recipients with LRI or at high risk for progression to RSV LRI (e.g., palivizumab 15 mg/kg body weight) (Hirsch et al., 2013).

Moreover, several agents are under development against RSV infections (Shahani et al., 2017). Firstly, there are fusion inhibitors like GS-5806 which reduced the viral load and the severity of clinical disease in a study of healthy adults (DeVincenzo et al., 2014), MDT-637^5^ and ALX-0171. Secondly, agents targeting the RSV polymerase exist: AL-8176 which had an interesting antiviral activity compared to placebo in 62 healthy adults inoculated with RSV (DeVincenzo et al., 2015) and favipiravir which is currently being tested in phase III clinical trials in the United States, Europe, and Latin America. Thirdly, substances targeting the viral nucleocapsid protein have been investigated. A small interfering RNA called ALN RSV01 inhibiting the synthesis of the RSV nucleocapsid protein has demonstrated a benefit in phase I to IIb clinical trials by decreasing the infection rate, reducing symptoms and the incidence of bronchiolitis obliterans. Another drug, RSV-604, targets the N terminal portion of the nucleocapsid protein. It was tested on HCT recipients, however, no data have been published (Simões et al., 2015). Finally, polyclonal high-titer anti RSV antibodies might be an option (RI-001)^6^.

Current strategies recommended for the treatment of respiratory viral disease after HCT, and molecules that are under investigation, are summarized in Table 1.

**Table 1:** Treatment algorithm of respiratory viral infections after HCT.

(1) Steroid dose withdrawal (if applicable) below 1 mg/kg/day (except for IFV)						
↓						
(2) In case of LRI or high risk factors: start specific treatment						
↓						
Virus	RSV	PIV	HMPV	RhV	AdV	IFV
First-line treatment recommendations	Oral ribavirin 30 mg/kg/day in 3 divided doses, 10 days				IV Cidofovir 5 mg/kg once a week (2w) and 5 mg/kg once every fortnight	Oral oseltamivir 75 mg bid (30 mg bid 10–15 kg, 45 mg bid 16–23 kg,. 60mg bid 24-40 kg) for 10 days
Alternative treatment	+IVIG IV ribavirin Inhaled ribavirin Palivizumab (young children)				Oral brincidofovir (2mg/kg twice a week] Anti-ADV CTL	NAI: inhaled zanamivir IV peramivir Zanamivir (if resistance) M2 ion channel inhibitor: amantadine (IV-A)
Drugs/immunotherapy in development	GS-5806 MDT-637 ALX-0171 Favipiravir RSV-604 AL-8176 ALN-RSV01 RI-001	DAS 181 Anti PIV-3 CTL	MAb 338	Oral/nasal pleconaril vapendavir	HBX	Inhaled laninamivir Nitazoxanide MEDI8852 VIS410
↓						
**(3) Virus spread prophylaxis: hand hygiene, surgical mask, vaccination of family, prolonged isolation**						

*AdV, adenovirus; HMPV, human metapneumovirus; RhV, rhinovirus; IFV, influenza virus ; IVIG, intravenous polyvalent immunoglobulins ; LRI, lower respiratory tract infection; NAI, neuraminidase inhibitor; PIV, parainfluenza virus; RSV, respiratory syncytial virus.*

### Immunotherapy After HCT

Before the era of specific antiviral manufactured T cells, donor lymphocyte infusions (DLI) were used and recommended in case of disseminated AdV infections after HCT (Bordigoni et al., 2001; Taniguchi et al., 2012). The high incidence of GvHD after DLIs limits its use in patients with previous history of GvHD. Anecdotal use of DLI has been reported for threatening RSV LRI (Kishi et al., 2000).

There are currently three strategies to manufacture viral specific T cells (VST) for clinical use: The first one is direct selection of antiviral T cells, either by multimers specific for a virus-derived peptide in the setting of class I HLA molecule, or by column selection of IFN-γ expressing T cells (with immunomagnetic beads) after viral antigen stimulation (Feuchtinger et al., 2006; Peggs et al., 2011; Icheva et al., 2013). The second one is *ex vivo* expansion of T cells co-cultured with antigen presenting cells pulsed, infected or transfected with viral peptide/protein/viral lysate or plasmid (Peggs et al., 2003; Gerdemann et al., 2012, 2013). The third one is genetic modification of T cells which incorporate high affinity VSTs receptor or chimeric antigen receptor genes (Schub et al., 2009; Cruz et al., 2013; Bollard and Heslop, 2016).

So far, in the setting of community acquired viral respiratory infections, adoptive immune therapy has been developed and investigated in clinical trials only for AdV infections. This strategy is promising as it has been reported to offer a way to cure severe adenoviral infections in case of resistance to antiviral drugs. Adenoviral clearance has been obtained after infusions of AdV-VST generated by interferon [IFN-γ-based immunomagnetic isolation (and further *in vivo* expansion) from initial donor or third party donor] in 10 of 11 hematopoietic stem cells transplanted patients in a French phase I/II trial (Qian et al., 2017). Similar results have been published earlier (Feucht et al., 2015) from 30 patients who received AdV-VST for AdV disease or viremia. 14 of 23 evaluable patients had *in vivo* expansion of Th1-VST (median time 24 days); and 21 patients responded to VST infusions and AdV clearance occurred in 14. Of note, AdV-VST infusions were not associated with acute toxicities or significant onset of GvHD. The main disadvantage of this technique is that high doses of immunosuppressive drugs and especially steroids should be avoided before infusions as this drug hampers *in vivo* expansion of specific T cells.

Currently, multiviral (including AdV) T cell manufacturing and banking from a third-party donor is a promising strategy as it might offer an immediate way to prevent AdV infections after HCT or to cure 71% of AdV infected patients after HCT (Tzannou et al., 2017). *Ex vivo* expanded cytotoxic T cells against PIV-3 antigens should be further tested in clinical trials (McLaughlin et al., 2016; Aguayo-Hiraldo et al., 2017).

### Outcomes and Later Monitoring

#### Overall Mortality and Risk Factors

The outcome in patients who experience progression to LRI is worse compared to patients with URI regardless of the virus involved (Chemaly et al., 2014). In a French cohort of 131 adult patients infected with viral respiratory infections, 16 (12%) died within 3 months. Among these, eight patients (6%) died of viral pneumonia along with bacterial and/or fungal pneumonia. Interestingly, the virus group and the time from HCT had no impact on mortality. Two factors were independently associated with increased overall mortality: steroid dose over 1 mg/kg body weight and a lymphocyte count lower than 0.5 g/L (Wolfromm et al., 2014). The fact that LRI associated death risk does not depend on the virus type is supported by Seo et al. (2017) who reported an overall mortality probability at day 90 as high as 41% in patients after HCT who developed RhV LRI, a rate that was comparable to RSV, PIV and IFV LRI in an adjusted model. Among children, in a cohort of 58 patients, 3 (5%) died of respiratory failure (Choi et al., 2013). LRI in the absence of URI and adenoviral infection has been described to be associated with a poorer outcome in another cohort of 166 patients with specific overall mortality of 7% (Lo et al., 2013).

#### Pulmonary Outcomes After Viral Infections

Bronchiolitis obliterans syndrome and obstructive airflow decline have been associated with PIV and RSV infections within the first 3 months after allogeneic HCT which persisted at 1 year after transplant (Versluys et al., 2010). Further, the occurrence of a LRI within 100 days post-transplant was an independent predictive factor for late onset non-infectious pulmonary complications diagnosed in the first 36 months after transplantation in an observational prospective cohort study on 198 patients (Bergeron et al., 2018). Therefore, functional pulmonary explorations and long-term surveillance are warranted after community acquired viral infections which occurred early after HCT. To better understand risk factors of pulmonary complications in children after HCT, a multicentric French trial is ongoing^7^. Early treatment with β2 mimetics and inhaled steroids might be useful and should be proposed in case of persistent obstructive syndrome after respiratory viral infections resolution in HCT recipients.

## Concluding Discussion

This review has limitations because we present results from small case series that have restricted meaningfulness. Further, it includes recommendations that were in part made before the era of T cell repleted haploidentical transplantations. This is an area of intense research and these new developments might impact the epidemiology and outcomes of viral infections after HCT.

Respiratory virus infections continue to cause disease both in the pre-transplant as well as in the post-transplant period and should be taken very seriously, especially in children who tend to shed virus for longer periods. This long shedding time has implications for infection control. Therefore, both personnel and patients and their families should be properly instructed in hand hygiene. In case of pre-transplant infections, HCT should be deferred but the underlying disease, the conditioning regimen and donor availability need to be considered. In pediatric patients who also tend to acquire viruses more frequently, we favor transplant delay in elected cases, even in asymptomatic patients.

Without doubt, disease progression from URI to LRI influences the outcome in HCT recipients. There are a number of risk factors shared by respiratory viruses that are associated with LRI occurrence; lymphopenia appears to be a specific risk factor in this case. Other risk factors shared with increased mortality are steroid use at the time of LRI diagnosis, oxygen requirement at the time of BAL, and severity of disease based on APACHE II score. In addition, myeloablative conditioning, GvHD, bacterial as well as fungal pulmonary co-infections (especially with IFV and PIV infections) seem to worsen the outcome.

Is there a possibility to reduce the risk of progression from URI to LRI? There might be a chance if co-infections can be reduced or diagnosed and treated promptly. These co-infections could be more easily avoided if they were nosocomially acquired by transfer from personnel or visitors. Therefore, people who have access to the patient should be aware of this problem. Also, shortening of the lymphopenic interval could reduce disease progression. This might be achieved with a stricter withdrawal of immunosuppressive drugs, e.g., steroids, or a milder form of conditioning.

Early and specific diagnostics are of the essence. It would be best to obtain diagnostic samples straight from the area of infection, i.e., from BAL material and not a nasal swab in case of a LRI, however, this might be sometimes impractical and not be tolerated by the patient. Unless a specific viral agent is suspected and a single or duplex PCR are ordered, e.g., during an outbreak, novel multiplex assays should be employed that also identify a bacterial co-infection. What is more, novel multiplex assays that are able to quantify viral loads clearly have the potential to help identify which patients are at increased risk and who might need treatment urgently – particularly since supporting measures like radiological imaging techniques often do not point to a specific pathogen and provide non-specific results with regard to identification of the pathogen responsible. However, while chest x-rays discover inflammatory processes with delay, CT findings might help to differentiate between pathogens. All these diagnostic steps should be carried out after full evaluation of the circumstances and in an orderly fashion. The ISI and the RSI (Tables 2, 3) might be helpful to identify patients at risk but await validation in clinical trials. Eventually, only few antivirals are available and licensed to treat respiratory virus infections. Several drugs have been tested in HCT recipients and promising results have been published. Novel antiviral compounds are urgently required that might be used in combination with antiviral lymphocytes as currently being developed for CMV, EBV, AdV, or BK antivirus CTLs in US (NCT01945814, NCT01570283) and European international trials (the TRACE trial). The production of these cells should be confined to specialized laboratories, either university-based or commercial, however, they should be easily accessible. The expansion of this approach to target respiratory viruses might be similarly useful to halt disease progression from URI to LRI.

**Table 2:** Immunodeficiency scoring index (ISI) for RSV.

Criteria	Weighing criteria	Assigned weights (score)
ANC < 500/μL	>2.5	3
ALC < 200/μL	>2.5	3
Age ≥ 40 years	2.0-2.5	2
Myeloablative conditioning regimen	<2.0	1
GVHD (acute or chronic)	<2.0	1
Corticosteroids within the last 30 days	<2.0	1
Recent or pre-engraftment allo-HCT	<2.0	1
Low risk: 0–2 score, moderate risk 3–6 score, high risk 7–12 score		

*ANC, absolute neutrophil count; ALC, absolute lymphocyte count; GVHD, graft-versus-host disease; Modified after Shah et al. (2014).*

**Table 3:** Scoring algorithm of the Radiologic Severity Index (RSI).

Lung zones (3 left lobe, 3 right lobe; 6 total) are evaluated by chest x-ray and CT scan; for each lobe there are three zones: (a) upper (above carina); (b) middle (below carina); (c) lower (below inferior pulmonary vein)			
**Pattern score**	**Predominant radiologic pattern in lung zone**	**Volumetric score**	**Extent of volumetric radiologic involvement**

1	Normal lung	0	0% (normal)
2	Ground-glass opacities	1	1–24%
3	Consolidation	2	25–49%
		3	50–74%
		4	75–100%

*To calculate the RSI score, the predominant pattern for each lung zone is multiplied with the extent of the volumetric radiologic involvement, e.g., if ground-glass opacities are detected in all six lung zones with all having a volumetric score of 3, the RSI score would be 36 (2 × 3 × 6). The maximum score is 72. Modified after Sheshadri et al. (2018).*

Finally, the application of CAR T cells to combat respiratory viral infections might be an option in the future, especially in combination with tumor targeting CAR T cells. However, pulmonary toxicity should be considered due to cross-reactivity of CAR T cells with non-targeted proteins, and also ARDS that often occurs in severe LRI might be worsened by cytokine release syndrome. While viral pneumonia still remains a concern in HCT patients, new drug developments, along with sophisticated T cell approaches, should diminish complications caused by respiratory viruses.

## Author Contributions

CP and SV wrote and edited the manuscript.

## Conflict of Interest Statement

The authors declare that the research was conducted in the absence of any commercial or financial relationships that could be construed as a potential conflict of interest.

## Footnotes

1 http://hadvwg.gmu.edu/

2 https://clinicaltrials.gov/ct2/show/NCT03481244

3 https://clinicaltrials.gov/ct2/show/NCT02367313

4 https://clinicaltrials.gov/ct2/show/NCT01502072

5 https://clinicaltrials.gov/ct2/show/NCT01556607

6 https://clinicaltrials.gov/ct2/show/NCT00632463

7 https://clinicaltrials.gov/ct2/show/NCT02032381

## Abbreviations

AdV: adenovirus
BAL: bronchoalveolar lavage
CMV: cytomegalovirus
GvHD: graft-*versus*-host disease
HBoV: human bocavirus
HCoV: human coronavirus
HCT: hematopoietic cell transplantation
HMPV: human metapneumovirus
IFV: influenza virus
ISI: immunodeficiency scoring index
IVIG: intravenous polyvalent immunoglobulins
LRI: lower respiratory tract infection
PCR: polymerase chain reaction
PIV: parainfluenza virus
RhV: rhinovirus
RSI: radiology severity index
RSV: respiratory syncytial virus
URI: upper respiratory tract infection

## References

[B1] Abandeh F. I. Lustberg M. Devine S. Elder P. Andritsos L. Martin S. I. (2013). Outcomes of hematopoietic SCT recipients with rhinovirus infection: a matched, case-control study. *Bone Marrow Transplant.* 48 1554–1557. 10.1038/bmt.2013.100 23872740 PMC4606879

[B2] Abbas S. Raybould J. E. Sastry S. de la Cruz O. (2017). Respiratory viruses in transplant recipients: more than just a cold. Clinical syndromes and infection prevention principles. *Int. J. Infect. Dis.* 62 86–93. 10.1016/j.ijid.2017.07.011 28739424

[B3] Abt M. C. Osborne L. C. Monticelli L. A. Doering T. A. Alenghat T. Sonnenberg G. F. (2012). Commensal bacteria calibrate the activation threshold of innate antiviral immunity. *Immunity* 37 158–170. 10.1016/j.immuni.2012.04.011 22705104 PMC3679670

[B4] Aguayo-Hiraldo P. I. Arasaratnam R. J. Tzannou I. Kuvalekar M. Lulla P. Naik S. (2017). Characterizing the cellular immune response to parainfluenza virus 3. *J. Infect. Dis.* 216 153–161. 10.1093/infdis/jix203 28472480 PMC5853958

[B5] Allander T. (2008). Human bocavirus. *J. Clin. Virol.* 41 29–33. 10.1016/j.jcv.2007.10.026 18055252

[B6] Allander T. Tammi M. T. Eriksson M. Bjerkner A. Tiveljung-Lindell A. Andersson B. (2005). Cloning of a human parvovirus by molecular screening of respiratory tract samples. *Proc. Natl. Acad. Sci. U.S.A.* 102 12891–12896. 10.1073/pnas.0504666102 16118271 PMC1200281

[B7] Arden K. E. McErlean P. Nissen M. D. Sloots T. P. Mackay I. M. (2006). Frequent detection of human rhinoviruses, paramyxoviruses, coronaviruses, and bocavirus during acute respiratory tract infections. *J. Med. Virol.* 78 1232–1240. 10.1002/jmv.20689 16847968 PMC7167201

[B8] Arthur J. L. Higgins G. D. Davidson G. P. Givney R. C. Ratcliff R. M. (2009). A novel bocavirus associated with acute gastroenteritis in Australian children. *PLoS Pathog.* 5:e1000391. 10.1371/journal.ppat.1000391 19381259 PMC2663820

[B9] Averbuch D. Safadi R. Dar D. Wolf D. Cherniak M. Sorek R. (2018). Successful brincidofovir treatment of metagenomics-detected adenovirus infection in a severely Ill STAT1-deficient patient. *Pediatr. Infect. Dis. J.* 10.1097/INF.0000000000002090 [Epub ahead of print]. 29742642

[B10] Avetisyan G. Mattsson J. Sparrelid E. Ljungman P. (2009). Respiratory syncytial virus infection in recipients of allogeneic stem-cell transplantation: a retrospective study of the incidence, clinical features, and outcome. *Transplantation* 88 1222–1226. 10.1097/TP.0b013e3181bb477e 19935377

[B11] Bergeron A. Chevret S. Peffault de Latour R. Chagnon K. de Margerie-Mellon C. Rivière F. (2018). Noninfectious lung complications after allogeneic haematopoietic stem cell transplantation. *Eur. Respir. J.* 51:1702617. 10.1183/13993003.02617-2017 29650555

[B12] Bicer S. Giray T. Col D. Erdag G. C. Vitrinel A. Gurol Y. (2013). Virological and clinical characterizations of respiratory infections in hospitalized children. *Ital. J. Pediatr.* 39:22. 10.1186/1824-7288-39-22 23536956 PMC3621398

[B13] Birger R. Morita H. Comito D. Filip I. Galanti M. Lane B. (2018). Asymptomatic shedding of respiratory virus among an ambulatory population across seasons. *mSphere* 3:e00667-18. 10.1128/mSphere.00249-18 29997120 PMC6041500

[B14] Boeckh M. (2008). The challenge of respiratory virus infections in hematopoietic cell transplant recipients. *Br. J. Haematol.* 143 455–467. 10.1111/j.1365-2141.2008.07295.x 18785968 PMC2735199

[B15] Boeckh M. Englund J. A. Li Y. Miller C. Cross A. Fernandez H. (2007). Randomized controlled multicenter trial of aerosolized ribavirin for respiratory syncytial virus upper respiratory tract infection in hematopoietic cell transplant recipients. *Clin. Inf. Dis.* 44 245–249. 10.1086/509930 17173225

[B16] Boivin G. Abed Y. Pelletier G. Ruel L. Moisan D. Cote S. (2002). Virological features and clinical manifestations associated with human metapneumovirus: a new paramyxovirus responsible for acute respiratory-tract infections in all age groups. *J. Infect. Dis.* 186 1330–1334. 10.1086/344319 12402203

[B17] Bollard C. M. Heslop H. E. (2016). T cells for viral infections after allogeneic hematopoietic stem cell transplant. *Blood* 127 3331–3340. 10.1182/blood-2016-01-628982 27207801 PMC4929925

[B18] Bordigoni P. Carret A. S. Venard V. Witz F. Le Faou A. (2001). Treatment of adenovirus infections in patients undergoing allogeneic hematopoietic stem cell transplantation. *Clin. Infect. Dis. Off. Publ. Infect. Dis. Soc. Am.* 32 1290–1297. 10.1086/319984 11303263

[B19] Boudreault A. A. Xie H. Leisenring W. Englund J. Corey L. Boeckh M. (2011). Impact of corticosteroid treatment and antiviral therapy on clinical outcomes in hematopoietic cell transplant patients infected with influenza virus. *Biol. Blood Marrow Transplant.* 17 979–986. 10.1016/j.bbmt.2010.09.014 20870025 PMC3676866

[B20] Bredius R. G. Templeton K. E. Scheltinga S. A. Claas E. C. Kroes A. C. Vossen J. M. (2004). Prospective study of respiratory viral infections in pediatric hemopoietic stem cell transplantation patients. *Pediatr. Infect. J.* 23 518–522. 10.1097/01.inf.0000125161.33843.bb 15194832

[B21] Campbell A. P. Guthrie K. A. Englund J. A. Farney R. M. Minerich E. L. Kuypers J. (2015). Clinical outcomes associated with respiratory virus detection before allogeneic hematopoietic stem cell transplant. *Clin. Infect. Dis. Off. Publ. Infect. Dis. Soc. Am.* 61 192–202. 10.1093/cid/civ272 25847977 PMC4565994

[B22] Casper C. Englund J. Boeckh M. (2010). How I treat influenza in patients with hematologic malignancies. *Blood* 115 1331–1342. 10.1182/blood-2009-11-255455 20009037 PMC2826758

[B23] Chang P. V. Hao L. Offermanns S. Medzhitov R. (2014). The microbial metabolite butyrate regulates intestinal macrophage function via histone deacetylase inhibition. *Proc. Natl. Acad. Sci. U.S.A.* 111 2247–2252. 10.1073/pnas.1322269111 24390544 PMC3926023

[B24] Chemaly R. F. Ghosh S. Bodey G. P. Rohatgi N. Safdar A. Keating M. J. (2006). Respiratory viral infections in adults with hematologic malignancies and human stem cell transplantation recipients: a retrospective study at a major cancer center. *Med. Baltim.* 85 278–287. 10.1097/01.md.0000232560.22098.4e 16974212

[B25] Chemaly R. F. Hanmod S. S. Rathod D. B. Ghantoji S. S. Jiang Y. Doshi A. (2012). The characteristics and outcomes of parainfluenza virus infections in 200 patients with leukemia or recipients of hematopoietic stem cell transplantation. *Blood* 119 2738–2745. 10.1182/blood-2011-08-371112 22246027

[B26] Chemaly R. F. Shah D. P. Boeckh M. J. (2014). Management of respiratory viral infections in hematopoietic cell transplant recipients and patients with hematologic malignancies. *Clin. Infect. Dis. Off. Publ. Infect. Dis. Soc. Am.* 59(Suppl. 5) S344–S351. 10.1093/cid/ciu623 25352629 PMC4303052

[B27] Ching W. Koyuncu E. Singh S. Arbelo-Roman C. Hartl B. Kremmer E. (2013). A ubiquitin-specific protease possesses a decisive role for adenovirus replication and oncogene-mediated transformation. *PLoS Pathog.* 9:e1003273. 10.1371/journal.ppat.1003273 23555268 PMC3610741

[B28] Choi J. H. Choi E. H. Kang H. J. Park K. D. Park S. S. Shin H. Y. (2013). Respiratory viral infections after hematopoietic stem cell transplantation in children. *J. Korean Med. Sci.* 28 36–41. 10.3346/jkms.2013.28.1.36 23341709 PMC3546101

[B29] Choi S.-M. Boudreault A. A. Xie H. Englund J. A. Corey L. Boeckh M. (2011). Differences in clinical outcomes after 2009 influenza A/H1N1 and seasonal influenza among hematopoietic cell transplant recipients. *Blood* 117 5050–5056. 10.1182/blood-2010-11-319186 21372154 PMC3109531

[B30] Choi S. M. Xie H. Campbell A. P. Kuypers J. Leisenring W. Boudreault A. A. (2012). Influenza viral RNA detection in blood as a marker to predict disease severity in hematopoietic cell transplant recipients. *J. Infect. Dis.* 206 1872–1877. 10.1093/infdis/jis610 23033148 PMC3502377

[B31] Chu H. Y. Renaud C. Ficken E. Thomson B. Kuypers J. Englund J. A. (2014). Respiratory tract infections due to human metapneumovirus in immunocompromised children. *J. Pediatr. Infect. Soc.* 3 286–293. 10.1093/jpids/piu100 25419459 PMC4240341

[B32] Cortez K. J. Erdman D. D. Peret T. C. Gill V. J. Childs R. Barrett A. J. (2001). Outbreak of human parainfluenza virus 3 infections in a hematopoietic stem cell transplant population. *J. Infect. Dis.* 184 1093–1097. 10.1086/322041 11598830

[B33] Cruz C. R. Y. Micklethwaite K. P. Savoldo B. Ramos C. A. Lam S. Ku S. (2013). Infusion of donor-derived CD19-redirected virus-specific T cells for B-cell malignancies relapsed after allogeneic stem cell transplant: a phase 1 study. *Blood* 122 2965–2973. 10.1182/blood-2013-06-506741 24030379 PMC3811171

[B34] de Lima C. R. Mirandolli T. B. Carneiro L. C. Tusset C. Romer C. M. Andreolla H. F. (2014). Prolonged respiratory viral shedding in transplant patients. *Transplant. Infect. Dis.* 16 165–169. 10.1111/tid.12167 24289829 PMC7169780

[B35] de Vries J. J. Bredius R. G. van Rheenen P. F. Smiers F. J. Scholvinck E. H. Vossen A. C. (2009). Human bocavirus in an immunocompromised child presenting with severe diarrhea. *J. Clin. Microbiol.* 47 1241–1243. 10.1128/JCM.01703-08 19193836 PMC2668299

[B36] Debiaggi M. Canducci F. Sampaolo M. Marinozzi M. C. Parea M. Terulla C. (2006). Persistent symptomless human metapneumovirus infection in hematopoietic stem cell transplant recipients. *J. Infect. Dis.* 194 474–478. 10.1086/505881 16845630

[B37] Debiaggi M. Canducci F. Terulla C. Sampaolo M. Marinozzi M. C. Alessandrino P. E. (2007). Long-term study on symptomless human metapneumovirus infection in hematopoietic stem cell transplant recipients. *New Microbiol.* 30 255–258. 17802904

[B38] Detweiler C. J. Mueller S. B. Sung A. D. Saullo J. L. Prasad V. K. Cardona D. M. (2018). Brincidofovir (CMX001) toxicity associated with epithelial apoptosis and crypt drop out in a hematopoietic cell transplant patient: challenges in distinguishing drug toxicity from GVHD. *J. Pediatr. Hematol. Oncol.* 40 e364–e368. 10.1097/MPH.0000000000001227 29846280 PMC6059994

[B39] DeVincenzo J. P. McClure M. W. Symons J. A. Fathi H. Westland C. Chanda S. (2015). Activity of oral ALS-008176 in a respiratory syncytial virus challenge study. *N. Engl. J. Med.* 373 2048–2058. 10.1056/NEJMoa1413275 26580997

[B40] DeVincenzo J. P. Whitley R. J. Mackman R. L. Scaglioni-Weinlich C. Harrison L. Farrell E. (2014). Oral GS-5806 activity in a respiratory syncytial virus challenge study. *N. Engl. J. Med.* 371 711–722. 10.1056/NEJMoa1401184 25140957

[B41] Dhakal B. D’Souza A. Pasquini M. Saber W. Fenske T. S. Moss R. B. (2016). DAS181 treatment of severe parainfluenza virus 3 pneumonia in allogeneic hematopoietic stem cell transplant recipients requiring mechanical ventilation. *Case Rep. Med.* 2016:8503275. 10.1155/2016/8503275 26941799 PMC4749780

[B42] Ditt V. Viazov S. Tillmann R. Schildgen V. Schildgen O. (2008). Genotyping of human bocavirus using a restriction length polymorphism. *Virus Genes* 36 67–69. 10.1007/s11262-007-0182-0 18071891 PMC7089024

[B43] Donohoe D. R. Garge N. Zhang X. Sun W. O’Connell T. M. Bunger M. K. (2011). The microbiome and butyrate regulate energy metabolism and autophagy in the mammalian colon. *Cell Metab.* 13 517–526. 10.1016/j.cmet.2011.02.018 21531334 PMC3099420

[B44] Dyer K. D. Drummond R. A. Rice T. A. Percopo C. M. Brenner T. A. Barisas D. A. (2016). Priming of the respiratory tract with immunobiotic *Lactobacillus plantarum* limits infection of alveolar macrophages with recombinant pneumonia virus of mice (rK2-PVM). *J. Virol.* 90 979–991. 10.1128/JVI.02279-15 26537680 PMC4702661

[B45] Edner N. Castillo-Rodas P. Falk L. Hedman K. Soderlund-Venermo M. Allander T. (2012). Life-threatening respiratory tract disease with human bocavirus-1 infection in a 4-year-old child. *J. Clin. Microbiol.* 50 531–532. 10.1128/JCM.05706-11 22135260 PMC3264148

[B46] Elemraid M. A. Muller M. Spencer D. A. Rushton S. P. Gorton R. Thomas M. F. (2014). Accuracy of the interpretation of chest radiographs for the diagnosis of paediatric pneumonia. *PLoS One* 9:e106051. 10.1371/journal.pone.0106051 25148361 PMC4141860

[B47] Engelhard D. Mohty B. de la Camara R. Cordonnier C. Ljungman P. (2013). European guidelines for prevention and management of influenza in hematopoietic stem cell transplantation and leukemia patients: summary of ECIL-4 (2011), on behalf of ECIL, a joint venture of EBMT, EORTC, ICHS, and ELN. *Transplant. Infect. Dis.* 15 219–32. 10.1111/tid.12054 23363310

[B48] Englund J. Feuchtinger T. Ljungman P. (2011). Viral infections in immunocompromised patients. *Biol. Blood Marrow Transplant.* 17 S2–S5. 10.1016/j.bbmt.2010.11.008 21195305 PMC3030455

[B49] Englund J. A. Boeckh M. Kuypers J. Nichols W. G. Hackman R. C. Morrow R. (2006). Brief communication: fatal human metapneumovirus infection in stem-cell transplant recipients. *Ann. Int. Med.* 144 344–349. 10.7326/0003-4819-144-5-200603070-00010 16520475

[B50] Englund J. A. Champlin R. E. Wyde P. R. Kantarjian H. Atmar R. L. Tarrand J. (1998). Common emergence of amantadine- and rimantadine-resistant influenza A viruses in symptomatic immunocompromised adults. *Clin. Infect. Dis. Off. Publ. Infect. Dis. Soc. Am.* 26 1418–1424. 10.1086/516358 9636873

[B51] Feghoul L. Chevret S. Cuinet A. Dalle J.-H. Ouachée M. Yacouben K. (2015). Adenovirus infection and disease in paediatric haematopoietic stem cell transplant patients: clues for antiviral pre-emptive treatment. *Clin. Microbiol. Infect. Off. Publ. Eur. Soc. Clin. Microbiol. Infect. Dis.* 21 701–709. 10.1016/j.cmi.2015.03.011 25882354

[B52] Feucht J. Opherk K. Lang P. Kayser S. Hartl L. Bethge W. (2015). Adoptive T-cell therapy with hexon-specific Th1 cells as a treatment of refractory adenovirus infection after HSCT. *Blood* 125 1986–1994. 10.1182/blood-2014-06-573725 25617426

[B53] Feuchtinger T. Lang P. Handgretinger R. (2007). Adenovirus infection after allogeneic stem cell transplantation. *Leuk Lymphoma* 48 244–255. 10.1080/10428190600881157 17325884

[B54] Feuchtinger T. Matthes-Martin S. Richard C. Lion T. Fuhrer M. Hamprecht K. (2006). Safe adoptive transfer of virus-specific T-cell immunity for the treatment of systemic adenovirus infection after allogeneic stem cell transplantation. *Br. J. Haematol.* 134 64–76. 10.1111/j.1365-2141.2006.06108.x 16803570

[B55] Fiore A. E. Fry A. Shay D. Gubareva L. Bresee J. S. Uyeki T. M. (2011). Antiviral agents for the treatment and chemoprophylaxis of influenza — recommendations of the advisory committee on immunization practices (ACIP). *MMWR Recomm. Rep. Morb. Mortal. Wkly. Rep. Recomm. Rep.* 60 1–24. 21248682

[B56] Franquet T. (2018). Imaging of community-acquired pneumonia. *J. Thorac Imaging.* 33 282–294. 10.1097/RTI.0000000000000347 30036297

[B57] Franquet T. Rodriguez S. Martino R. Gimenez A. Salinas T. Hidalgo A. (2006). Thin-section CT findings in hematopoietic stem cell transplantation recipients with respiratory virus pneumonia. *AJR Am. J. Roentgenol.* 187 1085–1090. 10.2214/AJR.05.0439 16985161

[B58] Gerdemann U. Katari U. L. Papadopoulou A. Keirnan J. M. Craddock J. A. Liu H. (2013). Safety and clinical efficacy of rapidly-generated trivirus-directed T cells as treatment for adenovirus, EBV, and CMV infections after allogeneic hematopoietic stem cell transplant. *Mol. Ther. J. Am. Soc. Gene Ther.* 21 2113–2121. 10.1038/mt.2013.151 23783429 PMC3831033

[B59] Gerdemann U. Keirnan J. M. Katari U. L. Yanagisawa R. Christin A. S. Huye L. E. (2012). Rapidly generated multivirus-specific cytotoxic T lymphocytes for the prophylaxis and treatment of viral infections. *Mol. Ther. J. Am. Soc. Gene Ther.* 20 1622–1632. 10.1038/mt.2012.130 22801446 PMC3412490

[B60] Gorcea C. M. Tholouli E. Turner A. Saif M. Davies E. Battersby E. (2017). Effective use of oral ribavirin for respiratory syncytial viral infections in allogeneic haematopoietic stem cell transplant recipients. *J. Hosp. Infect.* 95 214–217. 10.1016/j.jhin.2016.11.012 28077243

[B61] Grimley M. S. Chemaly R. F. Englund J. A. Kurtzberg J. Chittick G. Brundage T. M. (2017). Brincidofovir for asymptomatic adenovirus viremia in pediatric and adult allogeneic hematopoietic cell transplant recipients: a randomized placebo-controlled phase II trial. *Biol. Blood Marrow Transplant.* 23 512–521. 10.1016/j.bbmt.2016.12.621 28063938

[B62] Gutman J. A. Peck A. J. Kuypers J. Boeckh M. (2007). Rhinovirus as a cause of fatal lower respiratory tract infection in adult stem cell transplantation patients: a report of two cases. *Bone Marrow Transplant.* 40 809–811. 10.1038/sj.bmt.1705827 17704788 PMC7091740

[B63] Haak B. W. Littmann E. R. Chaubard J. L. Pickard A. J. Fontana E. Adhi F. (2018). Impact of gut colonization with butyrate-producing microbiota on respiratory viral infection following allo-HCT. *Blood* 131 2978–2986. 10.1182/blood-2018-01-828996 29674425 PMC6024637

[B64] Hamelin M.-E. Gagnon C. Prince G. A. Kiener P. Suzich J. Ulbrandt N. (2010). Prophylactic and therapeutic benefits of a monoclonal antibody against the fusion protein of human metapneumovirus in a mouse model. *Antivir. Res.* 88 31–37. 10.1016/j.antiviral.2010.07.001 20619294

[B65] Herbst T. Van Deerlin V. M. Miller W. T. (2013). The CT appearance of lower respiratory infection due to parainfluenza virus in adults. *AJR Am. J. Roentgenol.* 201 550–554. 10.2214/AJR.12.9613 23971445

[B66] Hilmes M. A. Daniel Dunnavant F. Singh S. P. Ellis W. D. Payne D. C. Zhu Y. (2017). Chest radiographic features of human metapneumovirus infection in pediatric patients. *Pediatr. Radiol.* 47 1745–1750. 10.1007/s00247-017-3943-5 28831577 PMC5901753

[B67] Hirsch H. H. Martino R. Ward K. N. Boeckh M. Einsele H. Ljungman P. (2013). Fourth European conference on infections in leukaemia (ECIL-4): guidelines for diagnosis and treatment of human respiratory syncytial virus, parainfluenza virus, metapneumovirus, rhinovirus, and coronavirus. *Clin. Infect. Dis.* 56 258–266. 10.1093/cid/cis844 23024295 PMC3526251

[B68] Hiwarkar P. Amrolia P. Sivaprakasam P. Lum S. H. Doss H. O’Rafferty C. (2017). Brincidofovir is highly efficacious in controlling adenoviremia in pediatric recipients of hematopoietic cell transplant. *Blood* 129 2033–2037. 10.1182/blood-2016-11-749721 28153824

[B69] Hiwarkar P. Kosulin K. Cesaro S. Mikulska M. Styczynski J. Wynn R. (2018). Management of adenovirus infection in patients after haematopoietic stem cell transplantation: state-of-the-art and real-life current approach: a position statement on behalf of the infectious diseases working party of the european society of blood and marrow transplantation. *Rev. Med. Virol.* 28:e1980. 10.1002/rmv.1980 29663594

[B70] Hodson A. Kasliwal M. Streetly M. MacMahon E. Raj K. (2011). A parainfluenza-3 outbreak in a SCT unit: sepsis with multi-organ failure and multiple co-pathogens are associated with increased mortality. *Bone Marrow Transplant.* 46 1545–1550. 10.1038/bmt.2010.347 21258418 PMC7091637

[B71] Hoellein A. Hecker J. Hoffmann D. Göttle F. Protzer U. Peschel C. (2016). Serious outbreak of human metapneumovirus in patients with hematologic malignancies. *Leuk. Lymphoma* 57 623–627. 10.3109/10428194.2015.1067699 26122193

[B72] Huang H. S. Tsai C. L. Chang J. Hsu T. C. Lin S. Lee C. C. (2017). Multiplex PCR system for the rapid diagnosis of respiratory virus infection: systematic review and meta-analysis. *Clin. Microbiol. Infect.* 24 1055–1063. 10.1016/j.cmi.2017.11.018 29208560 PMC7128951

[B73] Icheva V. Kayser S. Wolff D. Tuve S. Kyzirakos C. Bethge W. (2013). Adoptive transfer of epstein-barr virus (EBV) nuclear antigen 1-specific t cells as treatment for EBV reactivation and lymphoproliferative disorders after allogeneic stem-cell transplantation. *J. Clin. Oncol. Off. J. Am. Soc. Clin. Oncol.* 31 39–48. 10.1200/JCO.2011.39.8495 23169501

[B74] Ichinohe T. Pang I. K. Kumamoto Y. Peaper D. R. Ho J. H. Murray T. S. (2011). Microbiota regulates immune defense against respiratory tract influenza A virus infection. *Proc. Natl. Acad. Sci. U.S.A.* 108 5354–5359. 10.1073/pnas.1019378108 21402903 PMC3069176

[B75] Ison M. G. Hayden F. G. Kaiser L. Corey L. Boeckh M. (2003). Rhinovirus infections in hematopoietic stem cell transplant recipients with pneumonia. *Clin. Infect. Dis.* 36 1139–1143. 10.1086/374340 12715308 PMC7109670

[B76] Jacobs S. E. Soave R. Shore T. B. Satlin M. J. Schuetz A. N. Magro C. (2013). Human rhinovirus infections of the lower respiratory tract in hematopoietic stem cell transplant recipients. *Transplant. Infect. Dis.* 15 474–486. 10.1111/tid.12111 23890179 PMC3962254

[B77] Kanne J. P. Godwin J. D. Franquet T. Escuissato D. L. Muller N. L. (2007). Viral pneumonia after hematopoietic stem cell transplantation: high-resolution CT findings. *J. Thorac. Imaging* 22 292–299. 10.1097/RTI.0b013e31805467f4 17721347

[B78] Kapoor A. Hornig M. Asokan A. Williams B. Henriquez J. A. Lipkin W. I. (2011). Bocavirus episome in infected human tissue contains non-identical termini. *PLoS One* 6:e21362. 10.1371/journal.pone.0021362 21738642 PMC3125170

[B79] Kapoor A. Simmonds P. Slikas E. Li L. Bodhidatta L. Sethabutr O. (2010). Human bocaviruses are highly diverse, dispersed, recombination prone, and prevalent in enteric infections. *J. Infect. Dis.* 201 1633–1643. 10.1086/652416 20415538 PMC2902747

[B80] Kelly S. G. Metzger K. Bolon M. K. Silkaitis C. Mielnicki M. Cullen J. (2016). Respiratory syncytial virus outbreak on an adult stem cell transplant unit. *Am. J. Infect. Control* 44 1022–1026. 10.1016/j.ajic.2016.03.075 27430734

[B81] Khanna N. Steffen I. Studt J. D. Schreiber A. Lehmann T. Weisser M. (2009). Outcome of influenza infections in outpatients after allogeneic hematopoietic stem cell transplantation. *Transplant. Infect. Dis.* 11 100–105. 10.1111/j.1399-3062.2008.00362.x 19175540

[B82] Khanna N. Widmer A. F. Decker M. Steffen I. Halter J. Heim D. (2008). Respiratory syncytial virus infection in patients with hematological diseases: single-center study and review of the literature. *Clin. Infect. Dis.* 46 402–412. 10.1086/525263 18181739

[B83] Kim M. C. Kim M. Y. Lee H. J. Lee S. O. Choi S. H. Kim Y. S. (2016). CT findings in viral lower respiratory tract infections caused by parainfluenza virus, influenza virus and respiratory syncytial virus. *Med. Baltim.* 95:e4003. 10.1097/MD.0000000000004003 27368011 PMC4937925

[B84] Kim Y. J. Guthrie K. A. Waghmare A. Walsh E. E. Falsey A. R. Kuypers J. (2014). Respiratory syncytial virus in hematopoietic cell transplant recipients: factors determining progression to lower respiratory tract disease. *J. Infect. Dis.* 209 1195–1204. 10.1093/infdis/jit832 24368837 PMC3969549

[B85] Kim Y.-J. Waghmare A. Kuypers J. M. Jerome K. R. Pergam S. A. Xie H. (2017). Impact of pretransplant respiratory virus detection through universal screening in children undergoing hematopoietic cell transplantation (HCT). *Biol. Blood Marrow Transplant.* 23 S190–S191. 10.1016/j.bbmt.2016.12.370

[B86] Kishi Y. Kami M. Oki Y. Kazuyama Y. Kawabata M. Miyakoshi S. (2000). Donor lymphocyte infusion for treatment of life-threatening respiratory syncytial virus infection following bone marrow transplantation. *Bone Marrow Transplant.* 26 573–576. 10.1038/sj.bmt.1702559 11019850

[B87] Kmeid J. Vanichanan J. Shah D. P. El Chaer F. Azzi J. Ariza-Heredia E. J. (2016). Outcomes of influenza infections in hematopoietic cell transplant recipients: application of an immunodeficiency scoring index. *Biol. Blood Marrow Transplant.* 22 542–548. 10.1016/j.bbmt.2015.11.015 26638804 PMC4753109

[B88] Kohlmeier J. E. Woodland D. L. (2009). Immunity to respiratory viruses. *Annu. Rev. Immunol.* 27 61–82. 10.1146/annurev.immunol.021908.132625 18954284

[B89] Koskenvuo M. Mottonen M. Waris M. Allander T. Salmi T. T. Ruuskanen O. (2008). Human bocavirus in children with acute lymphoblastic leukemia. *Eur. J. Pediatr.* 167 1011–1015. 10.1007/s00431-007-0631-8 18038236 PMC7086950

[B90] Kosulin K. Berkowitsch B. Matthes S. Pichler H. Lawitschka A. Potschger U. (2018a). Intestinal adenovirus shedding before allogeneic stem cell transplantation is a risk factor for invasive infection post-transplant. *EBioMedicine* 28 114–119. 10.1016/j.ebiom.2017.12.030 29339099 PMC5835548

[B91] Kosulin K. Lam E. Heim A. Dobner T. Rodriguez E. (2018b). Broad-spectrum antiviral activity of the deubiquitinase inhibitor HBX against human adenoviruses. *Antivir. Ther.* 23 475–483. 10.3851/IMP3230 29557344

[B92] Kupfer B. Vehreschild J. Cornely O. Kaiser R. Plum G. Viazov S. (2006). Severe pneumonia and human bocavirus in adult. *Emerg. Infect. Dis.* 12 1614–1616. 10.3201/eid1210.060520 17176591 PMC3290954

[B93] Lalayanni C. Sirigou A. Iskas M. Smias C. Sakellari I. Anagnostopoulos A. (2010). Outbreak of novel influenza A (H1N1) in an adult haematology department and haematopoietic cell transplantation unit: clinical presentation and outcome. *J. Infect.* 61 270–272. 10.1016/j.jinf.2010.06.013 20600296

[B94] Lankester A. C. Heemskerk B. Claas E. C. J. Schilham M. W. Beersma M. F. C. Bredius R. G. M. (2004). Effect of ribavirin on the plasma viral DNA load in patients with disseminating adenovirus infection. *Clin. Infect. Dis. Off. Publ. Infect. Dis. Soc. Am.* 38 1521–1525. 10.1086/420817 15156436

[B95] Leber A. L. Everhart K. Daly J. A. Hopper A. Harrington A. Schreckenberger P. (2018). Multicenter evaluation of biofire filmarray respiratory panel 2 for detection of viruses and bacteria in nasopharyngeal swab samples. *J. Clin. Microbiol.* 56:e01945-17. 10.1128/JCM.01945-17 29593057 PMC5971546

[B96] Leen A. M. Bollard C. M. Myers G. D. Rooney C. M. (2006). Adenoviral infections in hematopoietic stem cell transplantation. *Biol. Blood Marrow Transplant.* 12 243–251. 10.1016/j.bbmt.2005.10.024 16503493

[B97] Lindemans C. A. Leen A. M. Boelens J. J. (2010). How I treat adenovirus in hematopoietic stem cell transplant recipients. *Blood* 116 5476–5485. 10.1182/blood-2010-04-259291 20837781 PMC3031399

[B98] Lion T. (2014). Adenovirus infections in immunocompetent and immunocompromised patients. *Clin. Microbiol. Rev.* 27 441–462. 10.1128/CMR.00116-13 24982316 PMC4135893

[B99] Ljungman P. (1997). Respiratory virus infections in bone marrow transplant recipients: the European perspective. *Am. J. Med.* 102 44–47. 10.1016/S0002-9343(97)00010-7 10868142

[B100] Ljungman P. de la Camara R. Perez-Bercoff L. Abecasis M. Nieto Campuzano J. B. Cannata-Ortiz M. J. (2011). Outcome of pandemic H1N1 infections in hematopoietic stem cell transplant recipients. *Haematologica* 96 1231–1235. 10.3324/haematol.2011.041913 21546495 PMC3148919

[B101] Ljungman P. Ward K. N. Crooks B. N. Parker A. Martino R. Shaw P. J. (2001). Respiratory virus infections after stem cell transplantation: a prospective study from the infectious diseases working party of the European Group for blood and marrow transplantation. *Bone Marrow Transplant.* 28 479–484. 10.1038/sj.bmt.1703139 11593321

[B102] Lo M. S. Lee G. M. Gunawardane N. Burchett S. K. Lachenauer C. S. Lehmann L. E. (2013). The impact of RSV, adenovirus, influenza, and parainfluenza infection in pediatric patients receiving stem cell transplant, solid organ transplant, or cancer chemotherapy. *Pediatr. Transplant.* 17 133–143. 10.1111/petr.12022 23228170

[B103] Martin E. T. Kuypers J. McRoberts J. P. Englund J. A. Zerr D. M. (2015). Human bocavirus 1 primary infection and shedding in infants. *J. Infect. Dis.* 212 516–524. 10.1093/infdis/jiv044 25632039 PMC4539892

[B104] Martino R. Porras R. P. Rabella N. Williams J. V. Ramila E. Margall N. (2005). Prospective study of the incidence, clinical features, and outcome of symptomatic upper and lower respiratory tract infections by respiratory viruses in adult recipients of hematopoietic stem cell transplants for hematologic malignancies. *Biol. Blood Marrow Transplant.* 11 781–796. 10.1016/j.bbmt.2005.07.007 16182179 PMC3347977

[B105] Mayer J. L. Lehners N. Egerer G. Kauczor H. U. Heussel C. P. (2014). CT-morphological characterization of respiratory syncytial virus (RSV) pneumonia in immune-compromised adults. *Rofo* 186 686–692. 10.1055/s-0033-1356353 24557598

[B106] Maziarz R. T. Sridharan P. Slater S. Meyers G. Post M. Erdman D. D. (2010). Control of an outbreak of human parainfluenza virus 3 in hematopoietic stem cell transplant recipients. *Biol. Blood Marrow Transplant.* 16 192–198. 10.1016/j.bbmt.2009.09.014 19781656 PMC7172256

[B107] McLaughlin L. P. Lang H. Williams E. Wright K. E. Powell A. Cruz C. R. (2016). Human parainfluenza virus-3 can be targeted by rapidly ex vivo expanded T lymphocytes. *Cytotherapy* 18 1515–1524. 10.1016/j.jcyt.2016.08.010 27692559 PMC5096976

[B108] Meijer A. Lackenby A. Hungnes O. Lina B. van-der-Werf S. Schweiger B. (2009). Oseltamivir-resistant influenza virus A (H1N1), Europe, 2007-08 season. *Emerg. Infect. Dis.* 15 552–560. 10.3201/eid1504.181280 19331731 PMC2671453

[B109] Memoli M. J. Hrabal R. J. Hassantoufighi A. Eichelberger M. C. Taubenberger J. K. (2010). Rapid selection of oseltamivir- and peramivir-resistant pandemic H1N1 virus during therapy in 2 immunocompromised hosts. *Clin. Infect. Dis. Off. Publ. Infect. Dis. Soc. Am.* 50 1252–1255. 10.1086/651605 20345239 PMC2946636

[B110] Milano F. Campbell A. P. Guthrie K. A. Kuypers J. Englund J. A. Corey L. (2010). Human rhinovirus and coronavirus detection among allogeneic hematopoietic stem cell transplantation recipients. *Blood* 115 2088–2094. 10.1182/blood-2009-09-244152 20042728 PMC2837322

[B111] Moe N. Krokstad S. Stenseng I. H. Christensen A. Skanke L. H. Risnes K. R. (2017). Comparing human metapneumovirus and respiratory syncytial virus: viral co-detections, genotypes and risk factors for severe disease. *PLoS One* 12:e0170200. 10.1371/journal.pone.0170200 28095451 PMC5240941

[B112] Morfin F. Dupuis-Girod S. Mundweiler S. Falcon D. Carrington D. Sedlacek P. (2005). *In vitro* susceptibility of adenovirus to antiviral drugs is species-dependent. *Antivir. Ther.* 10 225–229. 15865216

[B113] Neofytos D. Ojha A. Mookerjee B. Wagner J. Filicko J. Ferber A. (2007). Treatment of adenovirus disease in stem cell transplant recipients with cidofovir. *Biol. Blood Marrow Transplant.* 13 74–81. 10.1016/j.bbmt.2006.08.040 17222755

[B114] Nichols W. G. Corey L. Gooley T. A. Davis C. Boeckh M. (2001a). Parainfluenza virus infections after hematopoietic stem cell transplantation: risk factors, response to antiviral therapy, and effect on transplant outcome. *Blood* 98 573–578. 10.1182/blood.V98.3.573 11468152

[B115] Nichols W. G. Gooley T. Boeckh M. (2001b). Community-acquired respiratory syncytial virus and parainfluenza virus infections after hematopoietic stem cell transplantation: the fred hutchinson cancer research center experience. *Biol. Blood Marrow Transplant.* 7 11S–15S. 10.1053/bbmt.2001.v7.pm11777098 11777098

[B116] Nichols W. G. Erdman D. D. Han A. Zukerman C. Corey L. Boeckh M. (2004a). Prolonged outbreak of human parainfluenza virus 3 infection in a stem cell transplant outpatient department: insights from molecular epidemiologic analysis. *Biol. Blood Marrow Transplant.* 10 58–64. 10.1016/j.bbmt.2003.09.010 14752780

[B117] Nichols W. G. Guthrie K. A. Corey L. Boeckh M. (2004b). Influenza infections after hematopoietic stem cell transplantation: risk factors, mortality, and the effect of antiviral therapy. *Clin. Inf. Dis.* 39 1300–1306. 10.1086/425004 15494906

[B118] Ogimi C. Greninger A. L. Waghmare A. A. Kuypers J. M. Shean R. C. Xie H. (2017a). Prolonged shedding of human coronavirus in hematopoietic cell transplant recipients: risk factors and viral genome evolution. *J. Infect. Dis.* 216 203–209. 10.1093/infdis/jix264 28838146 PMC5853311

[B119] Ogimi C. Waghmare A. A. Kuypers J. M. Xie H. Yeung C. C. Leisenring W. M. (2017b). Clinical significance of human coronavirus in bronchoalveolar lavage samples from hematopoietic cell transplant recipients and patients with hematologic malignancies. *Clin. Infect. Dis.* 64 1532–1539. 10.1093/cid/cix160 28329354 PMC5434339

[B120] Ogimi C. Krantz E. M. Golob J. L. Waghmare A. Liu C. Leisenring W. M. (2018a). Antibiotic exposure prior to respiratory viral infection is associated with progression to lower respiratory tract disease in allogeneic hematopoietic cell transplant recipients. *Biol. Blood Marrow Transplant.* 24 2293–2301. 10.1016/j.bbmt.2018.05.016 29777867 PMC6286157

[B121] Ogimi C. Xie H. Leisenring W. M. Kuypers J. M. Jerome K. R. Campbell A. P. (2018b). Initial high viral load is associated with prolonged shedding of human rhinovirus in allogeneic hematopoietic cell transplant recipients. *Biol. Blood Marrow Transplant.* 24 2160–2163. 10.1016/j.bbmt.2018.07.006 30009982 PMC6239940

[B122] Parody R. Rabella N. Martino R. Otegui M. del Cuerpo M. Coll P. (2007). Upper and lower respiratory tract infections by human enterovirus and rhinovirus in adult patients with hematological malignancies. *Am. J. Hematol.* 82 807–811. 10.1002/ajh.20974 17563077

[B123] Peck A. J. Corey L. Boeckh M. (2004). Pretransplantation respiratory syncytial virus infection: impact of a strategy to delay transplantation. *Clin. Infect. Dis.* 39 673–680. 10.1086/422994 15356782

[B124] Peck A. J. Englund J. A. Kuypers J. Guthrie K. A. Corey L. Morrow R. (2007). Respiratory virus infection among hematopoietic cell transplant recipients: evidence for asymptomatic parainfluenza virus infection. *Blood* 110 1681–1688. 10.1182/blood-2006-12-060343 17502457 PMC1975849

[B125] Peggs K. S. Thomson K. Samuel E. Dyer G. Armoogum J. Chakraverty R. (2011). Directly selected cytomegalovirus-reactive donor T cells confer rapid and safe systemic reconstitution of virus-specific immunity following stem cell transplantation. *Clin. Infect. Dis. Off. Publ. Infect. Dis. Soc. Am.* 52 49–57. 10.1093/cid/ciq042 21148519

[B126] Peggs K. S. Verfuerth S. Pizzey A. Khan N. Guiver M. Moss P. A. (2003). Adoptive cellular therapy for early cytomegalovirus infection after allogeneic stem-cell transplantation with virus-specific T-cell lines. *Lancet Lond. Engl.* 362 1375–1377. 10.1016/S0140-6736(03)14634-X 14585640

[B127] Pinana J. L. Gomez M. D. Perez A. Madrid S. Balaguer-Rosello A. Gimenez E. (2018). Community acquired respiratory virus lower respiratory tract disease in allogeneic stem cell transplantation recipient: risk factors and mortality from pulmonary virus-bacterial mixed infections. *Transplant. Infect. Dis.* 20:e12926. 10.1111/tid.12926 29809298 PMC7169706

[B128] Protheroe R. E. Kirkland K. E. Pearce R. M. Kaminaris K. Bloor A. Potter M. N. (2012). The clinical features and outcome of 2009 H1N1 influenza infection in allo-SCT patients: a British society of blood and marrow transplantation study. *Bone Marrow Transplant.* 47 88–94. 10.1038/bmt.2011.12 21358686

[B129] Qian C. Campidelli A. Wang Y. Cai H. Venard V. Jeulin H. (2017). Curative or pre-emptive adenovirus-specific T cell transfer from matched unrelated or third party haploidentical donors after HSCT, including UCB transplantations: a successful phase I/II multicenter clinical trial. *J. Hematol. Oncol.* 10:102. 10.1186/s13045-017-0469-0 28482908 PMC5421327

[B130] Ramsay I. D. Attwood C. Irish D. Griffiths P. D. Kyriakou C. Lowe D. M. (2017). Disseminated adenovirus infection after allogeneic stem cell transplant and the potential role of brincidofovir – Case series and 10 year experience of management in an adult transplant cohort. *J. Clin. Virol. Off. Publ. Pan Am. Soc. Clin. Virol.* 96 73–79. 10.1016/j.jcv.2017.09.013 29017084

[B131] Renaud C. Campbell A. P. (2011). Changing epidemiology of respiratory viral infections in hematopoietic cell transplant recipients and solid organ transplant recipients. *Curr. Opin. Infect. Dis.* 24 333–343. 10.1097/QCO.0b013e3283480440 21666460 PMC3210111

[B132] Renaud C. Englund J. A. (2012). Antiviral therapy of respiratory viruses in haematopoietic stem cell transplant recipients. *Antivir. Ther.* 17 175–191. 10.3851/IMP2060) 22311587

[B133] Renaud C. Xie H. Seo S. Kuypers J. Cent A. Corey L. (2013). Mortality rates of human metapneumovirus and respiratory syncytial virus lower respiratory tract infections in hematopoietic cell transplant recipients. *Biol. Blood Marrow Transplant.* 19 1220–1226. 10.1016/j.bbmt.2013.05.005 23680472 PMC3752411

[B134] Salvatore M. Satlin M. J. Jacobs S. E. Jenkins S. G. Schuetz A. N. Moss R. B. (2016). DAS181 for treatment of parainfluenza virus infections in hematopoietic stem cell transplant recipients at a single center. *Biol. Blood Marrow Transplant.* 22 965–970. 10.1016/j.bbmt.2016.02.011 26904972

[B135] Sam S. S. Caliendo A. M. Ingersoll J. Abdul-Ali D. Hill C. E. Kraft C. S. (2018). Evaluation of performance characteristics of panther fusion assays for detection of respiratory viruses from nasopharyngeal and lower respiratory tract specimens. *J. Clin. Microbiol.* 56:e00787-18. 10.1128/JCM.00787-18 29793965 PMC6062789

[B136] Schenk T. Strahm B. Kontny U. Hufnagel M. Neumann-Haefelin D. Falcone V. (2007). Disseminated bocavirus infection after stem cell transplant. *Emerg. Infect. Dis.* 13 1425–1427. 10.3201/eid1309.070318 18252130 PMC2857292

[B137] Schiffer J. T. Kirby K. Sandmaier B. Storb R. Corey L. Boeckh M. (2009). Timing and severity of community acquired respiratory virus infections after myeloablative versus non-myeloablative hematopoietic stem cell transplantation. *Haematologica* 94 1101–1108. 10.3324/haematol.2008.003186 19644142 PMC2719033

[B138] Schildgen O. Muller A. Allander T. Mackay I. M. Volz S. Kupfer B. (2008). Human bocavirus: passenger or pathogen in acute respiratory tract infections? *Clin. Microbiol. Rev.* 21 291–304. 10.1128/CMR.00030-07 18400798 PMC2292574

[B139] Schleuning M. Buxbaum-Conradi H. Jäger G. Kolb H.-J. (2004). Intravenous ribavirin for eradication of respiratory syncytial virus (RSV) and adenovirus isolates from the respiratory and/or gastrointestinal tract in recipients of allogeneic hematopoietic stem cell transplants. *Hematol. J. Off. J. Eur. Haematol. Assoc.* 5 135–144. 10.1038/sj.thj.6200358 15048064

[B140] Schmidt M. E. Varga S. M. (2018). The CD8 T cell response to respiratory virus infections. *Front. Immunol.* 9:678. 10.3389/fimmu.2018.00678 29686673 PMC5900024

[B141] Schub A. Schuster I. G. Hammerschmidt W. Moosmann A. (2009). CMV-specific TCR-transgenic T cells for immunotherapy. *J. Immunol. Baltim. Md.* 1950 6819–6830. 10.4049/jimmunol.0902233 19864595

[B142] Semple M. G. Cowell A. Dove W. Greensill J. McNamara P. S. Halfhide C. (2005). Dual infection of infants by human metapneumovirus and human respiratory syncytial virus is strongly associated with severe bronchiolitis. *J. Infect. Dis.* 191 382–386. 10.1086/426457 15633097 PMC7109698

[B143] Senior K. (2002). FDA panel rejects common cold treatment. *Lancet Infect. Dis.* 2:264. 10.1016/S1473-3099(02)00277-3 12062983

[B144] Seo S. Campbell A. P. Xie H. Chien J. W. Leisenring W. M. Englund J. A. (2013). Outcome of respiratory syncytial virus lower respiratory tract disease in hematopoietic cell transplant recipients receiving aerosolized ribavirin: significance of stem cell source and oxygen requirement. *Biol. Blood Marrow Transplant.* 19 589–596. 10.1016/j.bbmt.2012.12.019 23298855 PMC3667608

[B145] Seo S. Gooley T. A. Kuypers J. M. Stednick Z. Jerome K. R. Englund J. A. (2016). Human metapneumovirus infections following hematopoietic cell transplantation: factors associated with disease progression. *Clin. Infect. Dis. Off. Publ. Infect. Dis. Soc. Am.* 63 178–185. 10.1093/cid/ciw284 27143659 PMC4928387

[B146] Seo S. Waghmare A. Scott E. M. Xie H. Kuypers J. M. Hackman R. C. (2017). Human rhinovirus detection in the lower respiratory tract of hematopoietic cell transplant recipients: association with mortality. *Haematologica* 102 1120–1130. 10.3324/haematol.2016.153767 28183847 PMC5451345

[B147] Seo S. Xie H. Campbell A. P. Kuypers J. M. Leisenring W. M. Englund J. A. (2014a). Parainfluenza virus lower respiratory tract disease after hematopoietic cell transplant: viral detection in the lung predicts outcome. *Clin. Infect. Dis. Off. Publ. Infect. Dis. Soc. Am.* 58 1357–1368. 10.1093/cid/ciu134 24599766 PMC4001290

[B148] Seo S. Xie H. Karron R. A. Thumar B. Englund J. A. Leisenring W. M. (2014b). Parainfluenza virus type 3 Ab in allogeneic hematopoietic cell transplant recipients: factors influencing post-transplant Ab titers and associated outcomes. *Bone Marrow Transplant.* 49 1205–1211. 10.1038/bmt.2014.124 24978141 PMC4332699

[B149] Shah D. P. Ghantoji S. S. Ariza-Heredia E. J. Shah J. N. El Taoum K. K. Shah P. K. (2014). Immunodeficiency scoring index to predict poor outcomes in hematopoietic cell transplant recipients with RSV infections. *Blood* 123 3263–3268. 10.1182/blood-2013-12-541359 24700783 PMC4046424

[B150] Shah D. P. Ghantoji S. S. Shah J. N. El Taoum K. K. Jiang Y. Popat U. (2013). Impact of aerosolized ribavirin on mortality in 280 allogeneic haematopoietic stem cell transplant recipients with respiratory syncytial virus infections. *J. Antimicrob. Chemother.* 68 1872–1880. 10.1093/jac/dkt111 23572228 PMC6296322

[B151] Shah D. P. Shah P. K. Azzi J. M. Chemaly R. F. (2016a). Parainfluenza virus infections in hematopoietic cell transplant recipients and hematologic malignancy patients: a systematic review. *Cancer Lett.* 370 358–364. 10.1016/j.canlet.2015.11.014 26582658 PMC4684719

[B152] Shah D. P. Shah P. K. Azzi J. M. El Chaer F. Chemaly R. F. (2016b). Human metapneumovirus infections in hematopoietic cell transplant recipients and hematologic malignancy patients: a systematic review. *Cancer Lett.* 379 100–106. 10.1016/j.canlet.2016.05.035 27260872 PMC4935561

[B153] Shah J. N. Chemaly R. F. (2011). Management of RSV infections in adult recipients of hematopoietic stem cell transplantation. *Blood* 117 2755–2763. 10.1182/blood-2010-08-263400 21139081

[B154] Shahani L. Ariza-Heredia E. J. Chemaly R. F. (2017). Antiviral therapy for respiratory viral infections in immunocompromised patients. *Expert Rev. Antivir. Infect. Ther.* 15 401–415. 10.1080/14787210.2017.1279970 28067078 PMC7103713

[B155] Sheshadri A. Shah D. P. Godoy M. Erasmus J. J. Song J. Li L. (2018). Progression of the radiologic severity index predicts mortality in patients with parainfluenza virus-associated lower respiratory infections. *PLoS One* 13:e0197418. 10.1371/journal.pone.0197418 29771962 PMC5957350

[B156] Simões E. A. F. DeVincenzo J. P. Boeckh M. Bont L. Crowe J. E. Griffiths P. (2015). Challenges and opportunities in developing respiratory syncytial virus therapeutics. *J. Infect. Dis.* 211 S1–S20. 10.1093/infdis/jiu828 25713060 PMC4345819

[B157] Spahr Y. Tschudin-Sutter S. Baettig V. Compagno F. Tamm M. Halter J. (2018). Community-acquired respiratory paramyxovirus infection after allogeneic hematopoietic cell transplantation: a single-center experience. *Open Forum Infect. Dis.* 5:ofy077. 10.1093/ofid/ofy077 29780847 PMC5952916

[B158] Srinivasan A. Wang C. Yang J. Shenep J. L. Leung W. H. Hayden R. T. (2011). Symptomatic parainfluenza virus infections in children undergoing hematopoietic stem cell transplantation. *Biol Blood Marrow Transplant.* 17 1520–1527. 10.1016/j.bbmt.2011.03.001 21396476 PMC4936785

[B159] Sung A. D. Sung J. A. M. Thomas S. Hyslop T. Gasparetto C. Long G. (2016). Universal mask usage for reduction of respiratory viral infections after stem cell transplant: a prospective trial. *Clin. Infect. Dis. Off. Publ. Infect. Dis. Soc. Am.* 63 999–1006. 10.1093/cid/ciw451 27481873 PMC5036914

[B160] Suyani E. Aki Z. Guzel O. Altindal S. Senol E. Sucak G. (2011). H1N1 infection in a cohort of hematopoietic stem cell transplant recipients: prompt antiviral therapy might be life saving. *Transplant. Infect. Dis.* 13 208–212. 10.1111/j.1399-3062.2010.00569.x 21214698

[B161] Syha R. Beck R. Hetzel J. Ketelsen D. Grosse U. Springer F. (2012). Human metapneumovirus (HMPV) associated pulmonary infections in immunocompromised adults–initial CT findings, disease course and comparison to respiratory-syncytial-virus (RSV) induced pulmonary infections. *Eur. J. Radiol.* 81 4173–4178. 10.1016/j.ejrad.2012.06.024 22795844

[B162] Taniguchi K. Yoshihara S. Tamaki H. Fujimoto T. Ikegame K. Kaida K. (2012). Incidence and treatment strategy for disseminated adenovirus disease after haploidentical stem cell transplantation. *Ann. Hematol.* 91 1305–1312. 10.1007/s00277-012-1440-3 22476883

[B163] Tomblyn M. Chiller T. Einsele H. Gress R. Sepkowitz K. Storek J. (2009). Guidelines for preventing infectious complications among hematopoietic cell transplantation recipients: a global perspective. *Biol. Blood Marrow Transplant.* 15 1143–1238. 10.1016/j.bbmt.2009.06.019 19747629 PMC3103296

[B164] Tzannou I. Papadopoulou A. Naik S. Leung K. Martinez C. A. Ramos C. A. (2017). Off-the-shelf virus-specific T cells to treat BK virus, human herpesvirus 6, cytomegalovirus, epstein-barr virus, and adenovirus infections after allogeneic hematopoietic stem-cell transplantation. *J. Clin. Oncol. Off. J. Am. Soc. Clin. Oncol.* 35 3547–3557. 10.1200/JCO.2017.73.0655 28783452 PMC5662844

[B165] Ulbrandt N. D. Ji H. Patel N. K. Riggs J. M. Brewah Y. A. Ready S. (2006). Isolation and characterization of monoclonal antibodies which neutralize human metapneumovirus *in vitro* and *in vivo*. *J. Virol.* 80 7799–7806. 10.1128/JVI.00318-06 16873237 PMC1563801

[B166] Ursic T. Steyer A. Kopriva S. Kalan G. Krivec U. Petrovec M. (2011). Human bocavirus as the cause of a life-threatening infection. *J. Clin. Microbiol.* 49 1179–1181. 10.1128/JCM.02362-10 21227992 PMC3067724

[B167] Ustun C. Slaby J. Shanley R. M. Vydra J. Smith A. R. Wagner J. E. (2012). Human parainfluenza virus infection after hematopoietic stem cell transplantation: risk factors, management, mortality, and changes over time. *Biol. Blood Marrow Transplant.* 18 1580–1588. 10.1016/j.bbmt.2012.04.012 22531491 PMC3443286

[B168] van den Hoogen B. G. de Jong J. C. Groen J. Kuiken T. de Groot R. Fouchier R. A. (2001). A newly discovered human pneumovirus isolated from young children with respiratory tract disease. *Nat. Med.* 7 719–724. 10.1038/89098 11385510 PMC7095854

[B169] van der Hoek L. Pyrc K. Jebbink M. F. Vermeulen-Oost W. Berkhout R. J. Wolthers K. C. (2004). Identification of a new human coronavirus. *Nat. Med.* 10 368–373. 10.1038/nm1024 15034574 PMC7095789

[B170] Versluys A. B. Rossen J. W. A. van Ewijk B. Schuurman R. Bierings M. B. Boelens J. J. (2010). Strong association between respiratory viral infection early after hematopoietic stem cell transplantation and the development of life-threatening acute and chronic alloimmune lung syndromes. *Biol. Blood Marrow Transplant.* 16 782–791. 10.1016/j.bbmt.2009.12.534 20060053 PMC7110441

[B171] von Lilienfeld-Toal M. Berger A. Christopeit M. Hentrich M. Heussel C. P. Kalkreuth J. (2016). Community acquired respiratory virus infections in cancer patients-guideline on diagnosis and management by the infectious diseases working party of the german society for haematology and medical oncology. *Eur. J. Cancer* 67 200–212. 10.1016/j.ejca.2016.08.015 27681877 PMC7125955

[B172] Vu D. Peck A. J. Nichols W. G. Varley C. Englund J. A. Corey L. (2007). Safety and tolerability of oseltamivir prophylaxis in hematopoietic stem cell transplant recipients: a retrospective case-control study. *Clin. Infect. Dis. Off. Publ. Infect. Dis. Soc. Am.* 45 187–193. 10.1086/518985 17578777

[B173] Waghmare A. Campbell A. P. Xie H. Seo S. Kuypers J. Leisenring W. (2013). Respiratory syncytial virus lower respiratory disease in hematopoietic cell transplant recipients: viral RNA detection in blood, antiviral treatment, and clinical outcomes. *Clin. Infect. Dis. Off. Publ. Infect. Dis. Soc. Am.* 57 1731–1741. 10.1093/cid/cit639 24065324 PMC3840404

[B174] Waghmare A. Englund J. A. Boeckh M. (2016). How I treat respiratory viral infections in the setting of intensive chemotherapy or hematopoietic cell transplantation. *Blood* 127 2682–2692. 10.1182/blood-2016-01-634873 26968533 PMC4891952

[B175] Waghmare A. Wagner T. Andrews R. Smith S. Kuypers J. Boeckh M. (2015). Successful treatment of parainfluenza virus respiratory tract infection with DAS181 in 4 immunocompromised children. *J. Pediatr. Infect. Soc.* 4 114–118. 10.1093/jpids/piu039 26185620 PMC4501511

[B176] Wang K. Wang W. Yan H. Ren P. Zhang J. Shen J. (2010). Correlation between bocavirus infection and humoral response, and co-infection with other respiratory viruses in children with acute respiratory infection. *J. Clin. Virol.* 47 148–155. 10.1016/j.jcv.2009.11.015 20022295 PMC7172221

[B177] Wen S. C. Williams J. V. (2015). New approaches for immunization and therapy against human metapneumovirus. *Clin. Vaccine Immunol.* 22 858–866. 10.1128/CVI.00230-15 26063237 PMC4519717

[B178] Whimbey E. Elting L. S. Couch R. B. Lo W. Williams L. Champlin R. E. (1994). Influenza A virus infections among hospitalized adult bone marrow transplant recipients. *Bone Marrow Transplant.* 13 437–440. 8019468

[B179] Williams J. V. Martino R. Rabella N. Otegui M. Parody R. Heck J. M. (2005). A prospective study comparing human metapneumovirus with other respiratory viruses in adults with hematologic malignancies and respiratory tract infections. *J. Infect. Dis.* 192 1061–1065. 10.1086/432732 16107960 PMC1483060

[B180] Wolfromm A. Porcher R. Legoff J. Peffault de Latour R. Xhaard A. (2014). Viral respiratory infections diagnosed by multiplex PCR after allogeneic hematopoietic stem cell transplantation: long-term incidence and outcome. *Biol. Blood Marrow Transplant.* 20 1238–1241. 10.1016/j.bbmt.2014.04.004 24732781 PMC7110602

[B181] Woo P. C. Lau S. K. Chu C. M. Chan K. H. Tsoi H. W. Huang Y. (2005). Characterization and complete genome sequence of a novel coronavirus, coronavirus HKU1, from patients with pneumonia. *J. Virol.* 79 884–895. 10.1128/JVI.79.2.884-895.2005 15613317 PMC538593

[B182] Ip W. W. Qasim W. (2013). Management of adenovirus in children after allogeneic hematopoietic stem cell transplantation. *Adv. Hematol.* 2013:176418. 10.1155/2013/176418 24288536 PMC3830830

[B183] Yan X. L. Li Y. N. Tang Y. J. Xie Z. P. Gao H. C. Yang X. M. (2017). Clinical characteristics and viral load of respiratory syncytial virus and human metapneumovirus in children hospitaled for acute lower respiratory tract infection. *J. Med. Virol.* 89 589–597. 10.1002/jmv.24687 27632796 PMC7166468

[B184] Yilmaz M. Chemaly R. F. Han X. Y. Thall P. F. Fox P. S. Tarrand J. J. (2013). Adenoviral infections in adult allogeneic hematopoietic SCT recipients: a single center experience. *Bone Marrow Transplant.* 48 1218–1223. 10.1038/bmt.2013.33 23503529 PMC4010139

